# Modelling the dynamics of tuberculosis lesions in a virtual lung: Role of the bronchial tree in endogenous reinfection

**DOI:** 10.1371/journal.pcbi.1007772

**Published:** 2020-05-20

**Authors:** Martí Català, Jordi Bechini, Montserrat Tenesa, Ricardo Pérez, Mariano Moya, Cristina Vilaplana, Joaquim Valls, Sergio Alonso, Daniel López, Pere-Joan Cardona, Clara Prats

**Affiliations:** 1 Comparative Medicine and Bioimage Centre of Catalonia (CMCiB), Fundació Institut d’Investigació en Ciències de la Salut Germans Trias i Pujol, Badalona, Catalonia, Spain; 2 Departament de Física, Universitat Politècnica de Catalunya, Castelldefels, Barcelona, Catalonia, Spain; 3 Servei de Radiodiagnòstic, Hospital Universitari Germans Trias i Pujol, Badalona, Catalonia, Spain; 4 Experimental Tuberculosis Unit, Fundació Institut d’Investigació en Ciències de la Salut Germans Trias i Pujol, Universitat Autònoma de Barcelona, Can Ruti Campus, Edifici Mar, Badalona, Catalonia, Spain; 5 Centro de Investigación Biomédica en Red de Enfermedades Respiratorias, Madrid, Spain; University of California Irvine, UNITED STATES

## Abstract

Tuberculosis (TB) is an infectious disease that still causes more than 1.5 million deaths annually. The World Health Organization estimates that around 30% of the world’s population is latently infected. However, the mechanisms responsible for 10% of this reserve (i.e., of the latently infected population) developing an active disease are not fully understood, yet. The dynamic hypothesis suggests that endogenous reinfection has an important role in maintaining latent infection. In order to examine this hypothesis for falsifiability, an agent-based model of growth, merging, and proliferation of TB lesions was implemented in a computational bronchial tree, built with an iterative algorithm for the generation of bronchial bifurcations and tubes applied inside a virtual 3D pulmonary surface. The computational model was fed and parameterized with computed tomography (CT) experimental data from 5 latently infected minipigs. First, we used CT images to reconstruct the virtual pulmonary surfaces where bronchial trees are built. Then, CT data about TB lesion’ size and location to each minipig were used in the parameterization process. The model’s outcome provides spatial and size distributions of TB lesions that successfully reproduced experimental data, thus reinforcing the role of the bronchial tree as the spatial structure triggering endogenous reinfection. A sensitivity analysis of the model shows that the final number of lesions is strongly related with the endogenous reinfection frequency and maximum growth rate of the lesions, while their mean diameter mainly depends on the spatial spreading of new lesions and the maximum radius. Finally, the model was used as an *in silico* experimental platform to explore the transition from latent infection to active disease, identifying two main triggering factors: a high inflammatory response and the combination of a moderate inflammatory response with a small breathing amplitude.

## Author summary

Tuberculosis is, even today, among the 10 main causes of death in the world. Despite the effectiveness of current strategies to fight the disease and those that are under development, the huge reservoir of latently infected individuals is a big hindrance in its eradication. One of the challenges inherent in this problem is that the mechanisms that cause latent infection to evolve towards active disease are not fully understood. Why will 90% of infected individuals never develop an active disease? In other words, what are the main factors that trigger an active disease in 10% of cases? We have focused our efforts on understanding the mechanisms that allow keeping infection latent, especially those related with endogenous reinfection. Since it is supposed to occur through the bronchial tree, we have designed a 3D computational model that mimics this structure, in which we have implemented an agent-based model of lesion growth and proliferation. Our results were contrasted with computed tomography measurements in latently infected minipigs, providing successful results that reinforce the essential role of endogenous reinfection through the bronchial tree in keeping infection latent.

## Introduction

Tuberculosis (TB) is an infectious disease that in 2017 killed more than 1.6 million people. *Mycobacterium tuberculosis* (*Mtb*) causes TB, and this bacterium is the individual agent causing the highest mortality worldwide [1]. The World Health Organization (WHO) estimates that 25 to 30% of the population worldwide is infected with *Mtb*, and that around 10% of infected people will develop active tuberculosis (ATB) in a few years’ time [2], although these percentages are being questioned and re-visited by recent studies [3]. WHO also estimates that 10 million humans developed ATB in 2017 [2].

**Fig 1:**
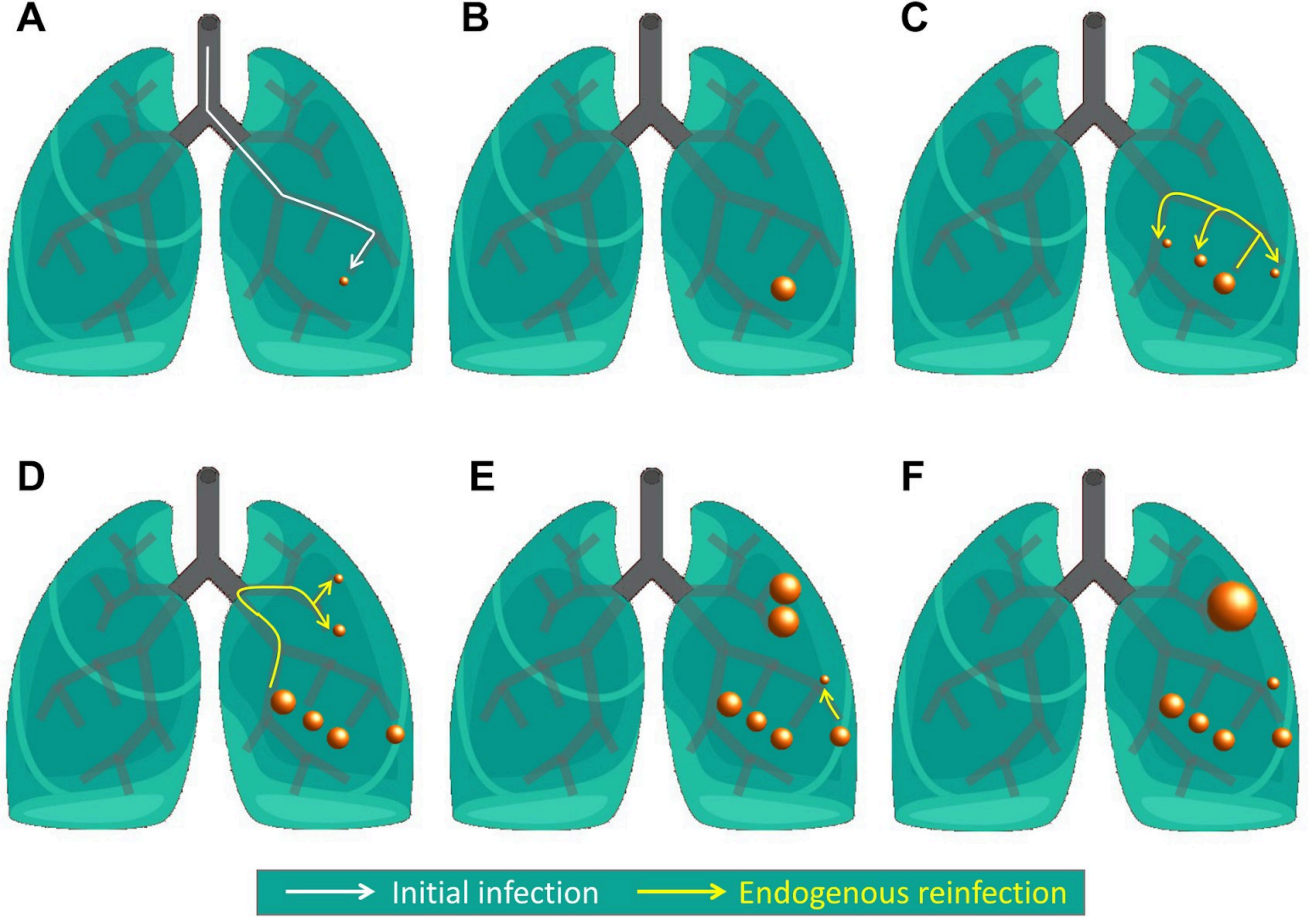
Main features of dynamic hypothesis. Schematic representation of initial infection (A, white arrow) lesions’ growth (A–F), endogenous reinfection (C–E, yellow arrows) and coalescence of neighbouring lesions (F).

TB infection starts at a pulmonary alveolus when *Mtb* is phagocyted by an alveolar macrophage (AM). The *Mtb* resists bactericidal mechanisms induced by AM and replicates inside the macrophage [4]. Under proper *in vitro* conditions *Mtb* replicates once a day [5]. When the intracellular bacterial load overcomes the AM’s maximum tolerability, macrophage necrosis is triggered, thereby returning bacilli to the extracellular milieu. These bacilli are phagocyted by other AMs and the cycle begins again giving rise to a further increase in bacilli. The further inclusion of more AMs fails to control bacillary growth. The death of AMs triggers a local inflammatory response first, and then a specific immune response, which finally controls the infection. The end of the progressive infection leaves an encapsulated TB lesion [6]. According to the dynamic hypothesis of Cardona [7], there is a certain probability that a few bacilli will escape from the lesion, mostly inside a foamy macrophage, and start a new infection in another alveolus. This process is assumed to occur through the bronchial tree, and it is what we denote as endogenous reinfection. This includes not only new infections generated from the initial infection site, but also those originated in successive infection foci (Fig 1). An *Mtb* infection may be completely cleared by the organism [8], it may enter into a latent state in which the host is infected but not sick and cannot infect other people, called Latent Tuberculosis Infection (LTBI), or, if the immune and inflammatory responses are not well balanced, the host may develop Active Tuberculosis disease (ATB).

Systems biology and computational models are fruitful tools for increasing understanding of the processes involved in TB [1]. Recently, different models have been useful in identifying several TB key factors [9–13]. In particular, the *Bubble model* suggests that the coalescence of closed lesions is the main mechanism for the growth of lesions in animals that progress to ATB [14]. This model successfully explains experimental observations in mice [15]. The *Bubble model* assumes a generalised logistic (Richard’s curve) [16] growth of lesions, driven by the inflammatory and immune responses, with their proliferation according to the endogenous reinfection theory, and a merging between neighbouring lesions when they are close enough. The model successfully reproduced ATB observed in C3HeB/FeJ mice, demonstrating the importance of local inflammation, lesion proliferation, and coalescence in the triggering of active disease. These results are relevant for mouse models; however, they are not easily extrapolated to humans, because of the differences between the structure of the lungs in the two species, in addition to the well-known differences in immune systems and encapsulation capacity.

Actually, the structure of the lungs may play an important role in the infection dynamics of TB. On the one hand, endogenous reinfection occurs mainly through the bronchial tree, and mice have much simpler pulmonary structure than humans, as no secondary lobular structure is found in mice (they have little or none interlobular septae) [17]. On the other hand, the encapsulation of lesions is driven by fibroblasts and fibrin from pulmonary membranes like intralobular septae. Nevertheless, mice do not possess intralobular septae and lesion encapsulation is not possible except close to the pleura [18]. In addition, the immune response of C3HeB/FeJ mice is much less effective than that of humans. In humans, a balanced Th1 immune response usually takes place after TB infection [19]. As mice’s immune response is not strong and encapsulation is not possible, these animal models cannot develop an LTBI situation and all experimental observations show ATB cases [15].

Although pigs and humans share a great deal of anatomy and physiology, researchers rarely employ pigs as *in vivo* models for TB. Yet their immune system and lung structure are particularly close to the corresponding system and structure in humans. Thus, TB development in pigs is more similar to that in humans than in mouse models [19, 20]. Minipigs are a genetically selected species, which is more convenient than other pigs for experiments in a lab, mainly for size reasons. Experimental results in TB in minipigs resemble pathological findings described in human [21–23].

In this study we aim to adapt and implement the *Bubble model* in a virtual bronchial tree in order to understand the maintenance of LTBI in minipigs. In particular, we want to test the falsifiability of the dynamic hypothesis of Cardona [7] that explains this maintenance, as well as to obtain some orders of magnitude of its dynamics. We use experimental minipig TB data to tune the model [23]. With the new model we perform several *in silico* experiments, which successfully reproduce experimental observations, and, furthermore, permit us to systematically explore the transition between ATB and LTBI.

In Materials and Methods we describe the CT experimental data, as well as the two sub-models used in simulations, which correspond to the computational lung and the revised Bubble model. We finish this section providing details of the model’s implementation and the methodology used for its parameterization and sensitivity analysis. Results’ section starts with an analysis of the computational lung obtained. Then, it provides the results of the model’s fitting to experimental data and the sensitivity analysis. Subsequent simulation series are used to explore the effect of the initial configuration and to test the transition between LTBI and ATB in minipigs. Finally, the conclusions for this study and their implications in testing the falsifiability of the dynamic hypothesis are drawn.

## Materials and methods

### Computer tomography measurements of LTBI in minipigs

Six female specific pathogen-free (spf) minipigs were intratracheally infected by H37Rv Pasteur strains of *Mtb* (10^3^ CFU) under sedation [23]. They were euthanized twelve weeks post-infection, without having received any TB treatment. None of them had developed ATB symptoms.

Broncho-pulmonary pieces were obtained from all minipigs and analysed with multidetector computed tomography scan (CT), a GE LightSpeed VCT with high image resolution (64-slice). The morphoanatomical study was carried out with volume rendering software. For each of the minipigs, we recorded the number of lesions, their location and size, Hounsfield units, and distance to closest pleura. One of the analysed minipigs did not present any lesions; it was considered non-infected for technical reasons and excluded from the subsequent analysis.

**Fig 2:**
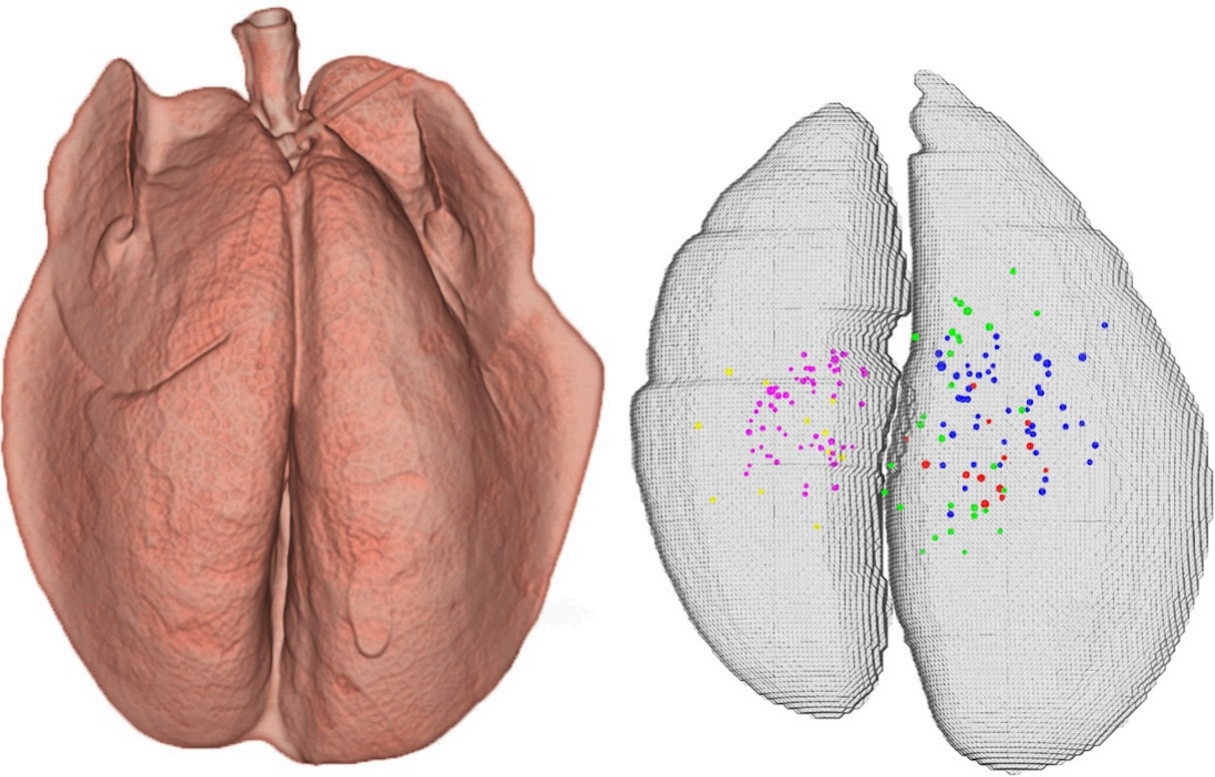
Summary of experimental results. Left: CT image reconstruction of a minipig’s pulmonary surface. Right: 3D representation of location and size of all minipig lesions; each colour is for a different minipig (red, magenta, blue, yellow, and green).

The 5 infected animals showed 165 lesions in total, 33 ± 22 per minipig. These lesions are shown in Fig 2. The mean diameter of the lesions was 1.3 ± 0.2 mm. Lesions were located in each minipig with a mean dispersion of 16 ± 4 mm. The number of lesions and their positions were used to train the computational model [24].

#### Ethics statement

All ethical requirements were followed according to Directive 201/63/EU, and the protocol and procedures of the study were approved by the corresponding ethical committee on animal welfare and the Catalan Government (Permit number: 5796). All animals were euthanized at week 12 post-infection by intravenous injection of sodium pentobarbital.

### Computational bronchial tree

The main novelty of this modelling approach is the use of an explicit 3D space that resembles a pulmonary bronchial tree. The design of this explicit space requires the building of a computational bronchial tree inside a certain pulmonary volume, limited by the external surface. The geometrical information necessary for this model can be obtained from pulmonary CT images of the studied minipigs.

Accordingly, the computational bronchial tree consists of two models: (1) an empirical model for the external lung’ surface that limits pulmonary volume, and (2) an artificial iterative model of bifurcations to build a bronchial tree inside this surface. This model is deterministic, since surfaces are obtained from experimental CT measurements and the iterative model does not incorporate randomness.

#### Empirical model for the lung’s external surface

An empirical surface model is built using CT scan data from one of the minipigs, randomly chosen. This representative surface is subsequently re-scaled according to the dimensions of others minipigs’ lungs, giving rise to 5 computational surfaces that can be used to buildi the 5 different bronchial trees.

**Fig 3:**
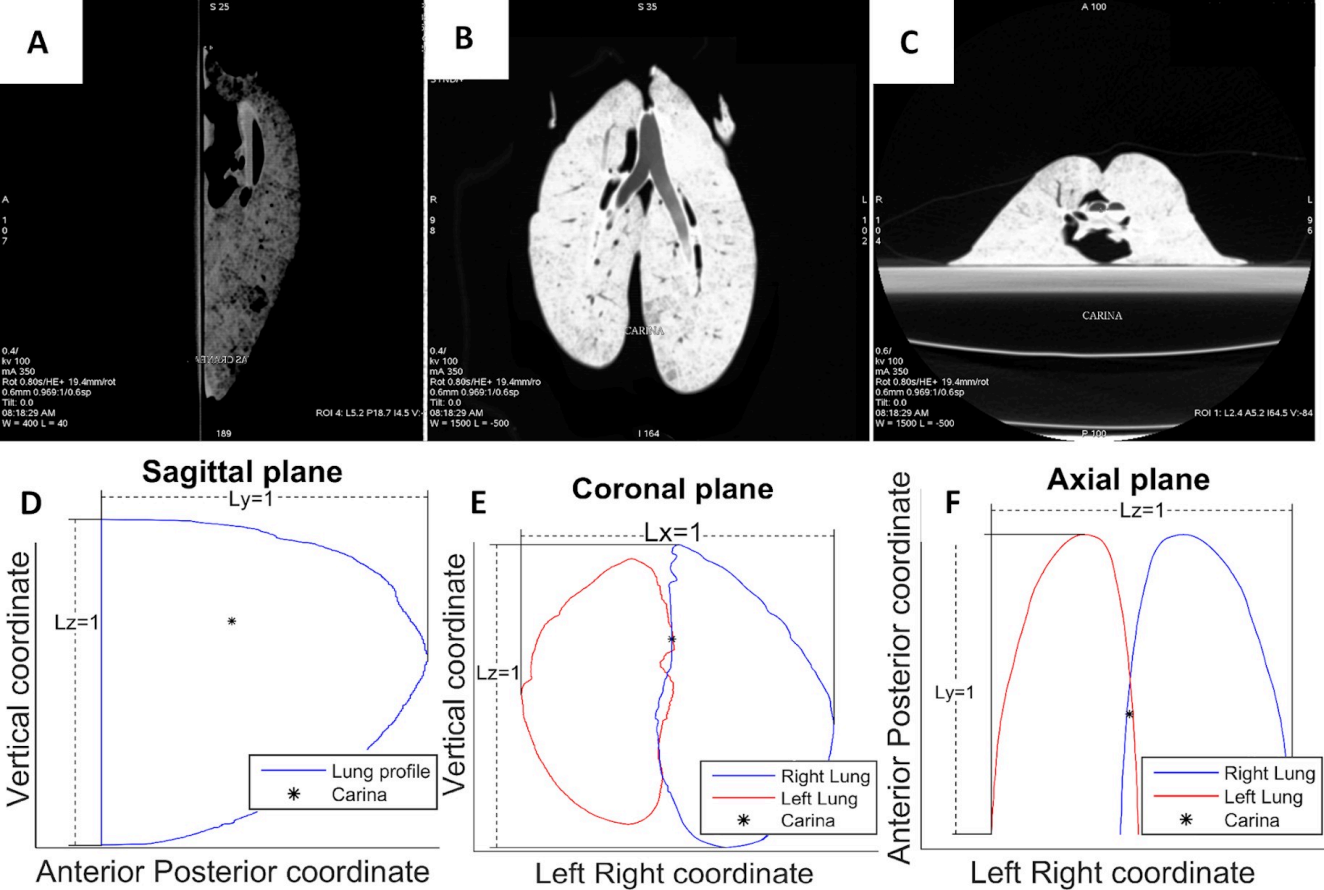
Normalized contour lines obtained from CT-scan images. Contour lines were obtained from CT-scan images and used for a 3D computational reconstruction of the pulmonary surface. Trachea division point (carina) is marked for purposes of reconstruction. (A) Sagittal plane CT image. (B) Coronal plane CT image. (C) Axial plane CT image. (D) Sagittal plane outline reconstruction. (E) Coronal plane outline reconstruction. (F) Axial plane outline reconstruction.

In order to build the representative pulmonary surface, we use three images of the three planes, i.e., coronal plane, sagittal plane, and axial plane. From these images, the contour line is extracted, keeping the carina (i.e., the bifurcation point of the trachea where it divides into the two main bronchi) position for purposes of reconstruction (Fig 3). The 3D reconstruction from contour lines is carried out with Matlab. All contours are normalized to 1 in order to be subsequently re-scaled with the specific dimensions of each of the 5 minipigs’ lungs on the reconstruction process. Finally, the left lung is slightly rotated (5°) so that the inter-pulmonary space is reduced.

All the lesions observed experimentally are located in the coordinate system defined by the reconstructions, taking the carina as the reference point, in order to check if they are located inside the obtained computational pulmonary surfaces. As a result, 95% of the detected lesions are inside the computed surface or in contact with pleura.

#### Iterative model for the bronchial tree

A bronchial tree of the conductive zone is built inside the computational pulmonary surface with an algorithm based on previous work on the human bronchial tree [25, 26]. Starting at the trachea, a set of iterative rules govern the successive bifurcations. The bronchial tree of the minipigs is assumed to be morphologically equivalent to the human one, but with smaller dimensions [27].

Our algorithm assumes that all the divisions are bifurcations, i.e., they occur in a dichotomous way. The resulting three branches involved in a bifurcation are coplanar, and the plane that contains each bifurcation is called a bifurcation plane. The divisions are assumed to occur in successive perpendicular planes, i.e., right-left, anterior-posterior, and upper-lower. Therefore, each bifurcation plane is perpendicular to the previous plane. The first division starts at the carina and directs the new branches into the right and left lungs.

When a certain conducting airway 0 divides into conducting airways 1 and 2, the flow conservation (*Q*_*0*_ *= Q*_*1*_ *+ Q*_*2*_) together with Murray’s law (*Q = C · d*^*3*^) [28] leads to the following relation between their diameters, *d*_*i*_:

$${d_{0}}^{3}={d_{1}}^{3}+{d_{2}}^{3}$$ (1)

Florens et al. [29] derived a ratio of 3 for the length of a branch (*l*_*i*_) and its diameter (*d*_*i*_) for most of the bronchial trees:

$$l_{i}=3d_{i}$$ (2)

We also studied this relation using Rozanek and Roubik’s experimental data [30], obtaining a proportionality constant of 3.07 and a goodness of fit of R^2^ = 0.98. This analysis is shown in the Supplementary material section 3 (Fig B in S1 File).

The diameters and angles of each bifurcation depend on the relative volume that each new branch supplies. We define the *q*_*i*_ factor as:

$$q_{i}=\frac{V_{i}}{V_{0}};i=1,2$$ (3)

where *V*_*1*_ and *V*_*2*_ are, respectively, the sub-volumes irrigated by conducting airways 1 and 2 after bifurcation, and *V*_*0*_ is the volume supplied by the branch 0 (before bifurcation). Taking into account Murray’s law, the diameters after the bifurcation are:

$$d_{i}=d_{0}\cdot {q_{i}}^{1/3};i=1,2$$ (4)

Minimizing the work per unit time (associated with friction and to maintain the structure) in bifurcations, the following relations between the angles of the bifurcation and the factor *q*_*i*_ are obtained [31]:

$$cos\phi_{i}=\frac{1}{2{q_{i}}^{2/3}}(1+{q_{i}}^{4/3}-{\left(1-q_{i}\right)}^{4/3});i=1,2$$ (5)

The calculation of the ratio *q*_*i*_ (Eq 3) cannot be analytically evaluated. Therefore, a grid of equispaced points is created so that the number of points inside each considered volume, *N*_*i*_ (*i = 0*, *1*, *2*), is assessed and the ratio is evaluated as:

$$q_{i}=\frac{N_{i}}{N_{0}};i=1,2$$ (6)

The distance between points is initially fixed at 1 mm [25] and then reduced to 0.2 mm to increase precision and improve results.

**Fig 4:**
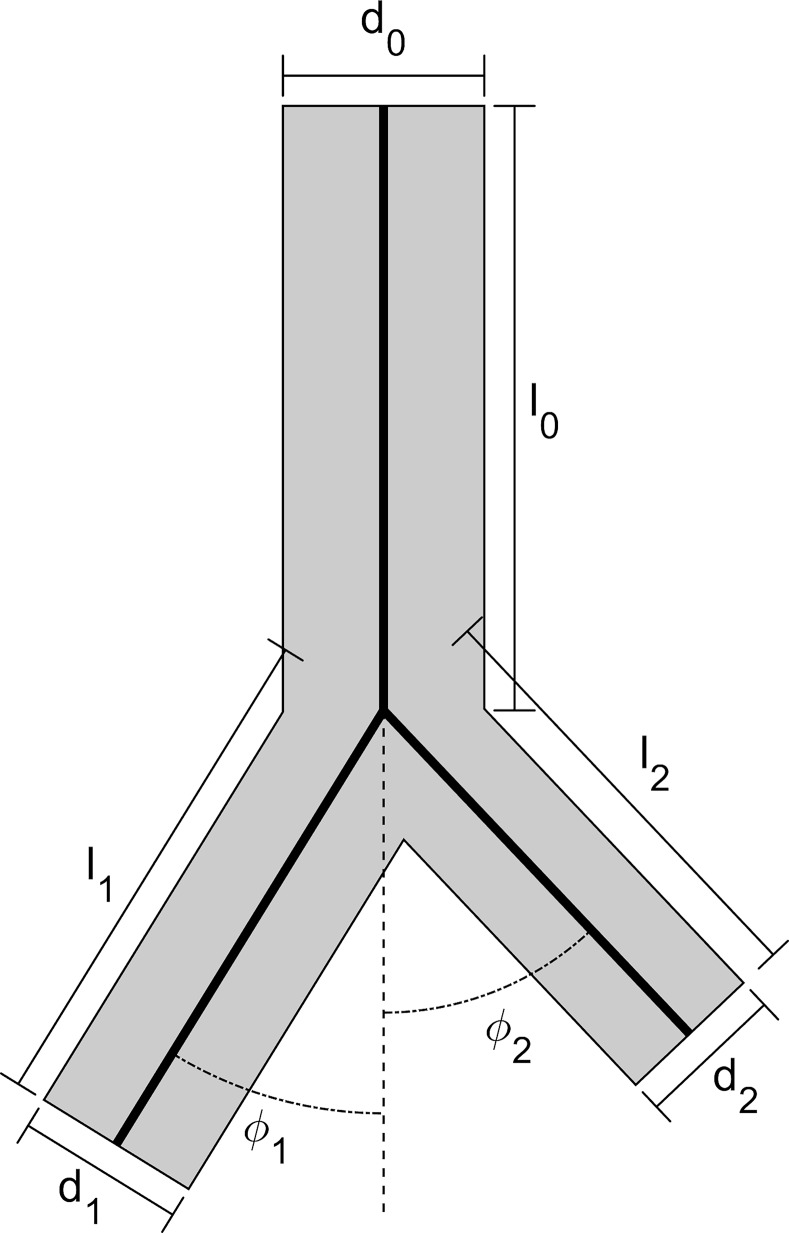
Bifurcation diagram. Bifurcation of 0 branch into two (1, 2) daughter branches. The cabal ratio for branch 1 is: q_1_ = 0.6. Length is 3 times the diameter of each branch as may be seen in Eq 2. Diameter relations are obtained from Eq 4, as d_1_ = 0.84·d_0_ and d_2_ = 0.74·d_0_. Angular values are computed using Eq 5, as ϕ_1_ = 32° and ϕ_2_ = 43°.

In Fig 4 there can be seen a diagram of a bifurcation example for q_1_ = 0.6. Using Eq 4 it can be determined that the diameter of the daughter branches are d_1_ = 0.84·d_0_ and d_2_ = 0.74·d_0_, respectively. Length is 3 times the diameter of each branch, then: l_0_ = 3·d_0_, l_1_ = 3·d_1_ = 2.53·d_0_ = 0.84·l_0_ and l_1_ = 3·d_1_ = 2.21·d_0_ = 0.74·l_0_. Bifurcation angles can be computed using Eq 5 as: ϕ_1_ = 32° and ϕ_2_ = 43°.

The initial tests with these equations resulted in a bronchial tree that does not fully occupy the upper part of the pulmonary surface. Therefore, a correction is added to the angle calculation [25]. It consists of an evaluation of the mass centre of the volumes to be supplied, ${\vec{x}}_{MC,V_{i}}$, which is projected on the bifurcation plane. We will denote with *ψ*_*i*_ the angle between the original branch and the projected mass centre. Experimentally, bifurcations with an angle greater than π/2 were not observed [32]. Therefore, the corrected angle *φ*_*i*_ is:

$$\varphi_{i}=min\left\{\frac{\phi_{i}+\psi_{i}}{2},\frac{\pi }{2}\right\};i=1,2$$ (7)

Branches with a diameter lower than 0.5 mm are considered terminals as areas those branches that escape from the pulmonary surface. The model for the building of successive bifurcations is summarized in Table 1.

**Table 1:** Summary of the model for building the computational bronchial tree of each minipig.

The normalized pulmonary surface is specifically re-scaled for each minipig, taking into account the measured dimensions.
A bronchial tree is built inside each computational pulmonary surface.
The bronchial tree starts at the end of the trachea, taking the trachea diameter and carina location of each minipig as reference.
The bronchial tree is a tubular structure, and non-terminal branches split in a dichotomous way.
The three branches implied in a division are coplanar (bifurcation plane).
In each division, the pulmonary territory is divided in two subregions by the plane that is perpendicular to the bifurcation plane, following the direction of the mother branch.
The bifurcation plane of the first division is the vertical one that separates the right and the left lungs.
The bifurcation planes are perpendicular from one generation to the following.
Once the bifurcation plane is defined, the ratio *q*_i_ = *V*_i_/*V*_0_ is numerically evaluated by a grid of equiespaced points with a precision of 0.2 mm, so that *q*_i_ = *N*_i_/*N*_0_ (*N*_i_ and *N*_0_ are the number of points contained in each subvolume).
The parameters of each bifurcation are evaluated using Eqs 2, 4, 5 and 7.
Branches that escape the pulmonary surface and those with a diameter of less than 0.5 mm are considered terminals.

### The *Bubble model*: an update

The *Bubble model* is an agent-based model in which the agents are the lesions. The dynamics of the lesions are driven by three processes: a generalised logistic growth, the reinfection process that permits the generation of new infection focuses, and the coalescence between neighbouring lesions.

The *Bubble model* was originally designed and calibrated to describe the dynamics of tuberculous lesions in mice with an active disease [14]. The spatial structure was not relevant for mice, due to the dimensions and the relatively simple structure of mouse lungs, and taking into consideration the size of lesions for the active disease. This model is updated and implemented as follows.

#### Lesion growth

We model a lesion as a sphere whose spatial position (3D coordinates, in mm), radius (in mm), and age (in days) are variables. Lesions are firstly detected when their radius is r_min_ = 0.075 mm (smaller lesions cannot be identified). This occurs after approximately t_min_ = 14 days from the initial infection. Then when a lesion is created it remains “silent” for 14 days before it is initialized with a r_min_ radius. The model employs a generalised logistic growth of the radius of the lesions as follows:

$$\frac{dr_{i}(t)}{dt}=v_{i}\cdot r_{i}(t)\cdot \left[1-{\left(\frac{r_{i}(t)}{r_{max}}\right)}^{2}\right]$$ (8)

where *r*_*i*_ is the radius of the lesion, *ν*_*i*_ is the parameter that sets the maximum growth rate, and *r*_max_ is the maximum radius. The parameter *ν*_*i*_ is modelled as a Gaussian variable with mean value *ν* and standard deviation ν/3. Therefore, each lesion grows at a slightly different velocity at each time step. From experimental data it is known that around the 28^th^ day a 2 mm lesion reaches its limit [22]; therefore, *ν* is estimated as *ν* = 0.3 day^-1^ (*σ*_*ν*_ = 0.1 day^-1^).

#### Lesion proliferation

**Fig 5:**
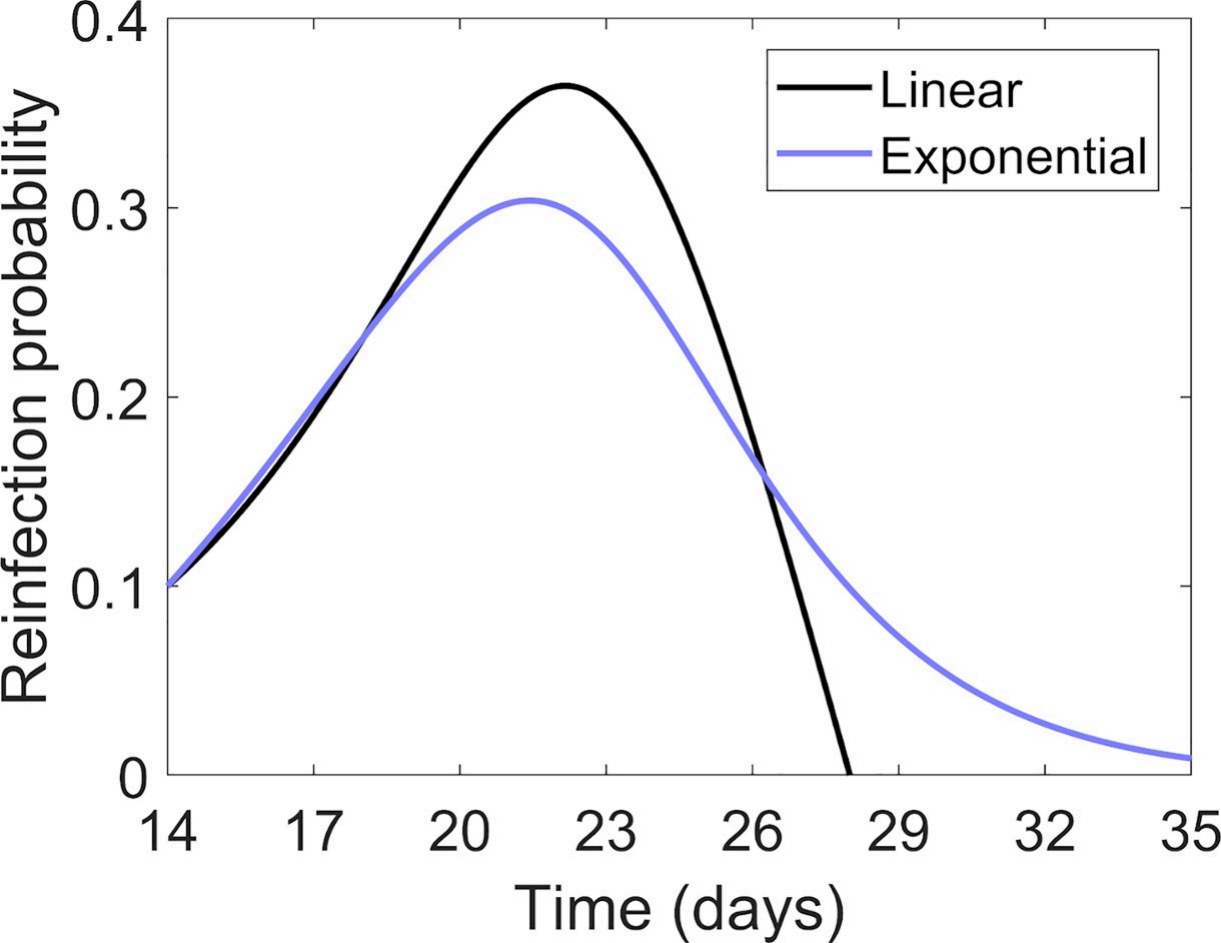
Reinfection probability. Reinfection probability for a lesion with *r*_max_ = 1 mm and *ρ* = 0.10 day^-1^. In black, original *Bubble model* [14]; in blue, the updated model considering an exponential decrease during the control phase. The area under the two curves is equivalent.

The multiplication of the number of lesions is caused by endogenous reinfection. In this way, a *mother* lesion generates new *daughter* lesions from day 14 to day 28. The original reinfection probability function [14] includes two terms: (1) a linearly increasing term with the radius of the mother, and (2) a linearly decreasing probability with *mother* lesion age [22]. The second term is slightly modified in order to allow the generation of new *daughter* lesions from *mother* lesions older than 28 days, with a small non-zero probability:

$$P(t)dt=\rho \cdot \frac{r_{i}(t)}{r_{min}}\cdot e^{-{\alpha (a_{i}-14)}^{n}}dt,a_{i}\geq 14days$$ (9)

where *ρ*, *α*, and *n* are parameters that define the probability profile, *a*_*i*_ is the age of the lesion, in days, and r_min_ is the minimum radius at which lesions are identified. Fig 5 shows original [14] and modified (Eq 9) models with *α* = 0.035 day^-n^ and *n* = 1.63 with *r*_max_ = 1 mm and *ρ* = 0.10 day^-1^. The values of *α* and *n* are fixed to ensure that the area under the two curves are equal and to minimize the difference between the experimental results and the linear model. The reinfection probability initially grows exponentially with the lesion radius; however, for longer times the curve decays exponentially because of encapsulation and calcification.

**Fig 6:**
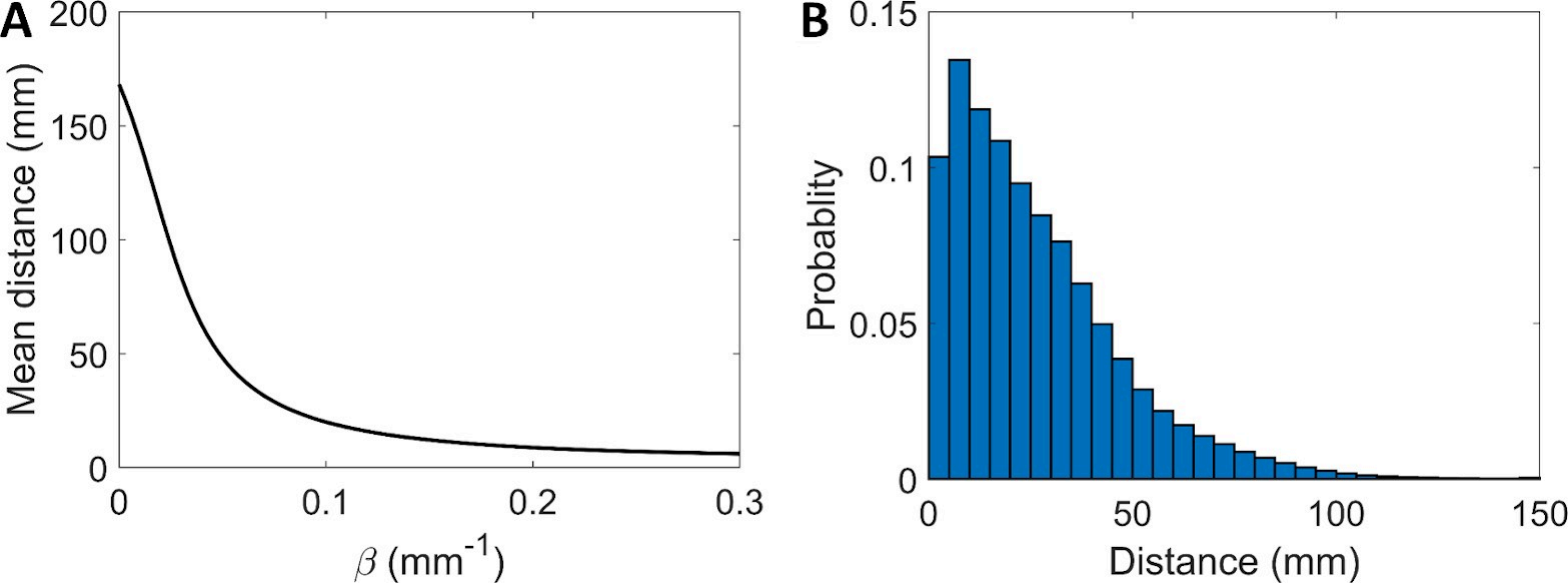
Lesion spreading distance. (A) Mean distance where lesions appear as a function of the dispersion parameter, *β* ∈ [0, 0.3] mm^-1^. Due to the quantization of the space, for *β* > 0.3 mm^-1^ the probability that the lesion appears at the nearest terminal is very high and the value is a constant. (B) Density of probability of new lesions spreading with *β* = 0.08 mm^-1^.

In the original model for mice, the location of new lesions is selected from a probability which decreases with the distance. In the current version for minipigs the distance is modelled in the same way, but considering the bronchial distance between two terminals instead of the geometric distance between two points. The model does not explicitly assume that the dissemination occurs exclusively through the aerial part, but it could include a possible recirculation through the adjacent circulatory or lymphatic systems as well. Then, possible locations are determined by the bronchial tree terminal positions:

$$P(i\to j)=\frac{e^{-\beta d_{ij}}}{\sum_{j}e^{-\beta d_{ij}}}$$ (10)

where *P* (*i → j*) is the probability that a lesion appears at a terminal *j* due to a *mother* lesion at terminal *i*, and *β* is the dispersion parameter that determines the spreading. Fig 6A shows the mean distance of the appearance of new lesions as a function of the dispersion parameter. Fig 6B shows the distribution of distance from daughter to mother lesions for *β* = 0.08 mm^-1^, which is slightly different from the theoretical distribution (Eq 10) due to spatial quantization.

#### Lesion coalescence

Coalescence occurs when the distance between two neighbouring lesions is shorter than the largest radius. Unlike the original model [14], the properties of the resulting lesion are weighted according to the coalescing lesions. The new radius is:

$${r_{new}}^{3}={r_{i}}^{3}+{r_{j}}^{3}$$ (11)

where *r*_*i*_ and *r*_*j*_ are the original radii of the two lesions. We employ the radius to calculate the weights:

$$\omega_{k}=\frac{r_{k}^{3}}{r_{new}^{3}};k=i,j$$ (12)

which are used to calculate the new age and position of the lesions

$$a_{new}=\omega_{i}\cdot a_{i}+\omega_{j}\cdot a_{j}$$ (13)

$${\vec{x}}_{new}=\omega_{i}\cdot {\vec{x}}_{i}+\omega_{j}\cdot {\vec{x}}_{j}$$ (14)

Coalescence process is considered every 10 time steps to reduce computational cost and to ensure that the results are not modified. There are no significant differences when one time step is used.

### Implementation and fitting

The computational models are implemented in Matlab. First, the bronchial trees of the 5 analysed minipigs are generated to obtain 5 virtual lungs. Next, we obtain the terminal locations and the distance between terminals through the bronchial tree. Finally, the evolution time of the lesions are calculated using the updated *Bubble model* implemented in the virtual lungs.

The model depends on a set of input parameters. It is not a deterministic model so the same set of parameters can have different outcomes. But there is a strong dependence between input parameters and outcome variables. Parameters *t*_min_, *r*_min_, *α*, *n*, and *ν* are adjusted as detailed above, according to the available information. The simulated total time, *T*_max_, is equal to the experimental time (i.e., 84 days). The number of time steps is fixed as 1000 in order to ensure model stability. There are three remaining parameters to be fitted:

- **Mean maximum radius, *r***_**max**_ is the mean radius achieved by non-merged lesions following a generalised logistic growth. It is measured in mm. To account for biology variability, each lesion has a different maximum radius. We use a Gaussian distribution of standard deviation *σ*_*r*max_ = 0.2 · *r*_max_ around the mean value.
- **Natality index, *ρ*** is proportional to the probability of triggering an endogenous reinfection process. It is measured in day^-1^.
- **Spreading parameter, *β*** determines spreading of the lesions through the bronchial tree due to endogenous reinfection process. It is measured in mm^-1^.

The complete model reproduces the evolution of an initial distribution of TB lesions in a 3D computational lung. Each single simulation consists of 5 independent runs, one for each virtual lung. The outcome results of the model are listed in Table 2.

**Table 2:** Outcome variables of the model.

Outcome variable	definition
Number of lesions	Number of lesions with a diameter greater than 0.9 mm (resolution threshold). These are called observable lesions.
Mean diameter	Mean diameter of the observable lesions. This is measured in mm.
Dispersion	Mean distance between observable lesions and their coordinates centre. This is measured in mm.
Coalescences	Number of merging processes that occurred during simulation.
Disease indicator	Logical index that indicates the presence or not of lesions larger than 10 mm.
Diameters histogram	Histogram of the diameter of the observable lesions. Edges of the histogram can be controlled.
Coordinates histogram	Histogram of the positions of the observable lesions. Edges of the histogram can be controlled.
Disease coordinates	Coordinates of the lesions larger than 10 mm. These are measured in mm taking the carina as origin.
Diameters dispersion	Standard deviation of the diameter of observable lesions. This is measured in mm.
Wounded volume	Total volume occupied by observable lesions. This is measured in cm^3^.
Time till disease	Elapsed simulation time till a larger lesion of 10 mm is found. This is measured in days.

#### Simulation scheduling

In order to assess the precision of the results obtained from the model, the variability of the different outcomes (Table 2) is measured. 25000 simulations are run to determine this variability. As mentioned above, in each simulation the model is run for the 5 virtual lungs that correspond to the 5 minipigs. A total of 125000 runs are done. This analysis is useful to learn whether the different results obtained with differing initial parameter simulations are significant or not.

The variability (error, *E*) of a given outcome, *OC*, is defined as:

$$E(OC,N)=\left|\frac{M(OC)-m(OC,N)}{M(OC)}\right|$$ (15)

where *m*(*OC*,*N*) is the mean value of a given outcome after performing *N* simulations and *M*(*OC*) is the limit of *m*(*OC*,*N*) when *N* is large enough, in this case *N* = 25000. The outcome values follow a Gaussian distribution, sometimes truncated due to quantization effects. Running simulations with different input parameters, *S* = {*ρ*, *β*, *r*_max_} and different number of simulations (*N*), a dependence between this variability (*E*), the targeted outcome (*OC*), the number of simulations (*N*), and the used set of parameters (*S*) is seen. This error is inversely proportional to the square root of the number of simulations. Then, Eq 15 can be rewritten as:

$$E(OC,N,S)=\left|\frac{M(OC,S)-m(OC,N,S)}{M(OC,S)}\right|=\frac{A(OC,S)}{\sqrt{N}}$$ (16)

where *A*(*OC*,*S*) is the proportionality constant that depends on the outcome variable (*OC*) and the given set of input parameters (*S*). Table 3 presents the values of *A*(*OC*,*S*) for different sets of parameters and outcomes.

**Table 3:** Values of proportionality constant, *A*(*OC*,*S*), for different outcomes and sets of input parameters.

		Latent set	Transition set	Active set
**Input parameters**	***ρ*** (day^-1^)	0.120	0.045	0.045
	***β*** (mm^-1^)	0.08	0.10	0.15
	***r***_***max***_ (mm)	0.68	6.50	10.00
**Proportionality constant**	**Number of lesions**	0.62	0.70	0.38
	**Mean diameter**	0.07	0.32	0.20
	**Dispersion**	0.10	0.88	3.38
	**Coalescences**	4.70	0.58	0.61
	**Disease percentage**	―――	0.58	―――

Values are approximated using *N* = 25000 simulations.

From this simulation schedule, we conclude that 500 simulations for each dataset ensures an error (variability) lower than 4% in most of the outcome values and different sets of parameters. Only in a few cases where the mean numeric value of the outcome is very small do we observe errors larger than 4%, but never larger than 20%.

#### Simplified protocol for model parameterization

We designed a protocol for parameterization, to find the set of parameters, *S* = {*ρ*, *β*, *r*_max_}, that best fit experimental data. We start our simulations with a single lesion at the mass centre of the experimentally observed distribution. To fit the three remaining parameters, we built three error functions to evaluate the agreement of three outcomes of the computer simulations with experimental data:

1. Error in number of lesions (*NLE*): relation of the number of lesions observed experimentally, *N*(*E*), and the final number of lesions obtained from a given simulation, *N*(*S*):
  $$NLE=\frac{\left|N(E)-N(S)\right|}{N(E)}$$ (17)
2. Error in distribution of diameters of the lesions (*DE*), relation of the diameter histogram, *HD*_*i*_(*E*), of the experimentally observed lesions with the corresponding simulation outcome (*HD*_*i*_(*S*)), *n*_*bins*_ being the number of bins in the histograms:
  $$DE=\frac{1}{N(E)}\sum_{i=1}^{n_{bins}}\left|{HD}_{i}(E)-{HD}_{i}(S)\frac{N(E)}{N(S)}\right|$$ (18)
3. Error in spatial location of lesions (*SE*), comparison of the experimental (*HS*_*i*,*j*_(*E*)) and the numerical (*HS*_*i*,*j*_(*S*) histograms of the spatial coordinates):
  $$SE=\frac{1}{3\cdot N(E)}\sum_{j=x,y,z}\sum_{i=1}^{n_{bins,j}}\left|{HS}_{i,j}(E)-{HS}_{i,j}(S)\frac{N(E)}{N(S)}\right|$$ (19)

These error functions will subsequently be used as objective functions to be minimized in the parameterization process.

The dependence between the objective functions and the input parameters is shown in Table 4. In this table, green means that the objective function is sensitive to this input parameter and that we can observe its minimum; orange means that it is sensitive but no minimum is observed; red means no sensitivity. Detailed plots are shown in supplementary material, section 4 (Fig C and Fig D, both in S1 File).

**Table 4:** Sensitivity of the error functions (NLE, DE, and SE) to the three parameters explored (*β*, *ρ* and *r*_max_).

Error function	*β*	*ρ*	*r*_max_
*Number of lesions error (NLE)*	IM	IM	IM
*Diameter error (DE)*	NM	NA	IM
*Spatial error (SE)*	IM	NA	NA

In green (IM), error functions that are affected by these parameters for which a minimum can be identified; in orange (NM), those that are affected but do not present a distinguishable minimum; in red (NA), those functions that are not affected by the parameter.

Given these results, we design the following process to fit the three parameters: (1) *β* is fitted by minimizing *SE*; (2) then, *r*_max_ is fitted by minimizing *DE*; (3) finally, *ρ* is fitted by minimizing *NLE*. This process is repeated iteratively to finally fit the three parameters jointly, because a change in one parameter can slightly move the minimum position of the three error functions.

#### Sensitivity analysis

The sensitivity analysis is performed using as initial infection a single lesion on the mass centre of the experimental lesions observed experimentally in each minipig. The sensitivity analysis is performed for 5 variables: *β*, *r*_max_, *ρ*, *ν*, and *T*_max_. A set of 11 simulations with different parameter values is designed: one simulation with all the parameters in their default values, five simulations increasing one-at-a-time parameter by 10%, and five simulations decreasing one-at-a-time parameter by 10%. We compute an ANOVA test analysis for 4 outcomes by comparing the results obtained with the original set of parameters and those obtained with each new parameter combination [33]. An extended sensitivity analysis is performed using [34] methodology; this sensitivity analysis is shown in Supplementary material section 2 (Fig A in S1 File).

## Results

### Computing the bronchial tree model: Properties and fitting with experimental data

**Fig 7:**
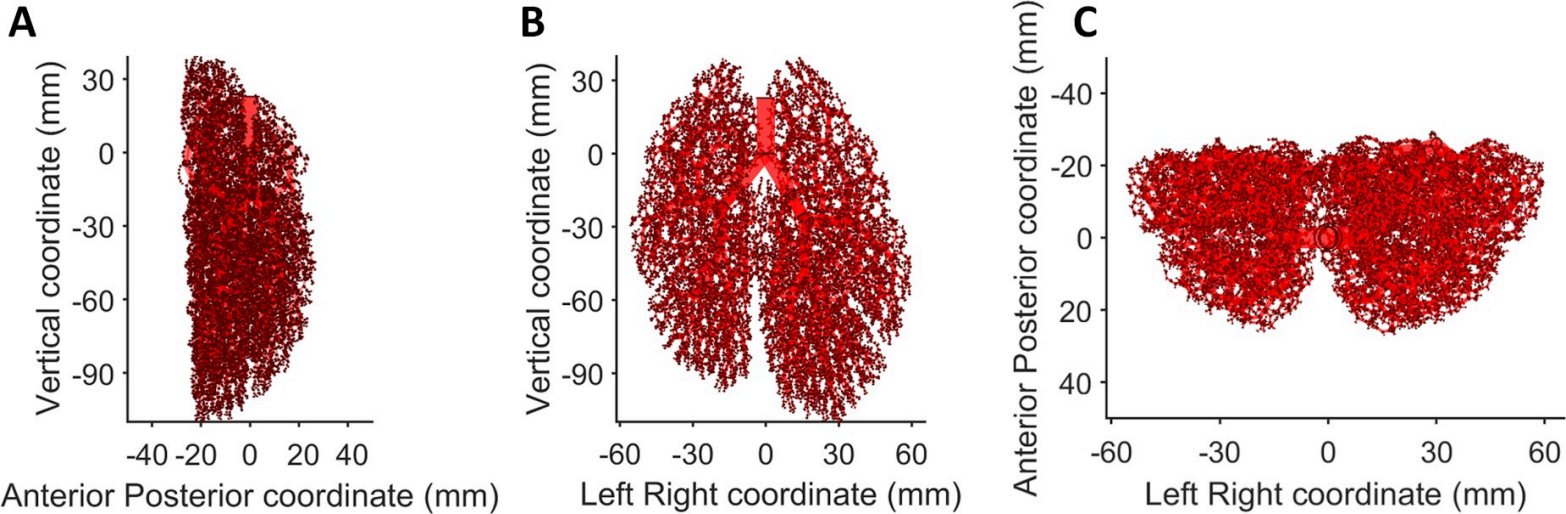
Computational minipig bronchial tree. It is represented as a tubular structure. Each conducting airway is represented by an empty cylinder. Three axial planes are shown: (A) Sagittal plane, (B) Coronal plane, and (C) Axial plane.

The surface of the minipig lungs is obtained from CT images. The 5 bronchial trees are generated inside the surfaces; see an example of computer generated bronchial tree in Fig 7. The largest tube is the trachea and at each division the alveolar diameter and length are reduced, according to the algorithm. The branches ramify and occupy all the pulmonary territory.

**Fig 8:**
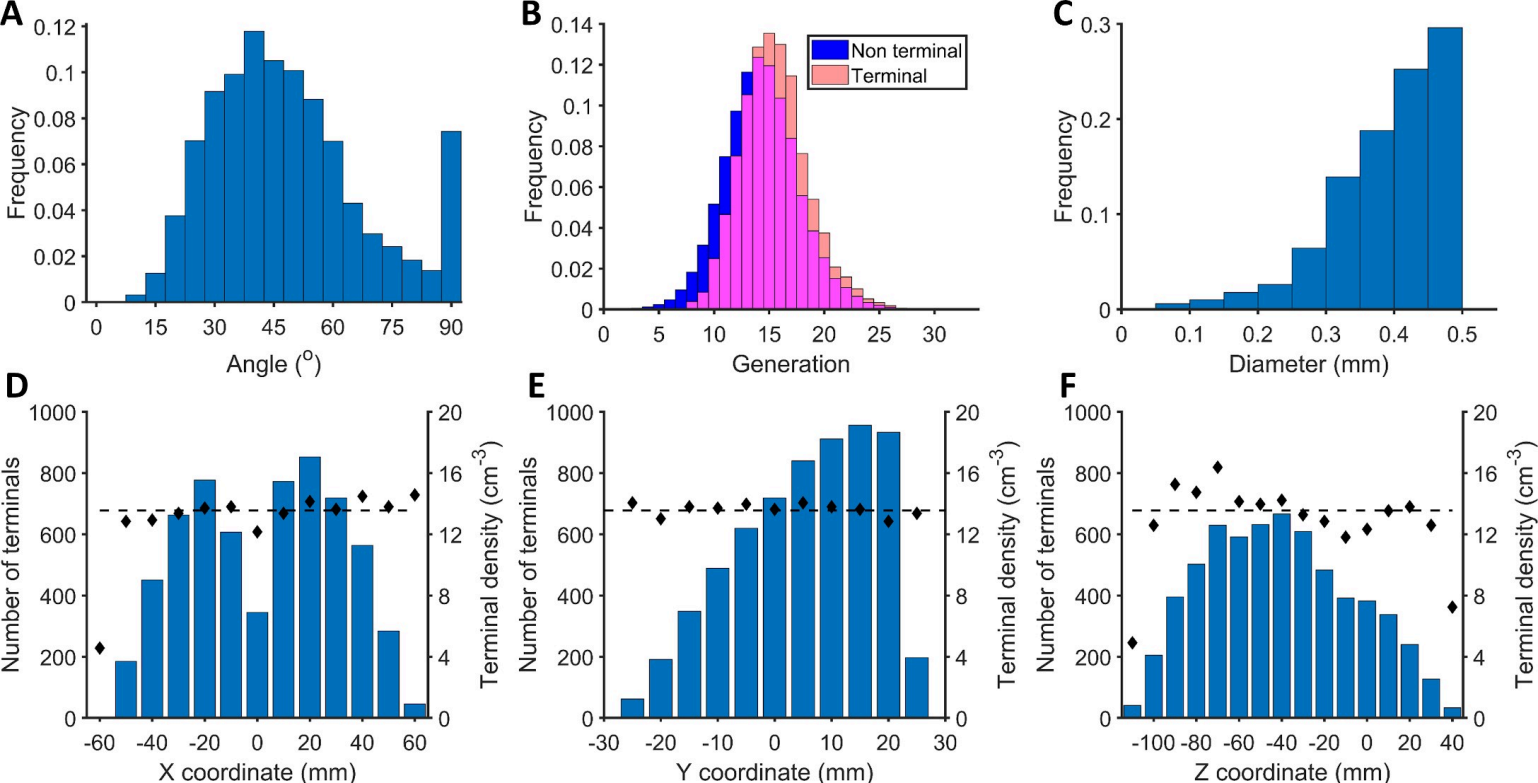
Computational bronchial tree analysis. (A) Angular distribution of bronchial tree bifurcations. The angle is measured between the mother branch and the new one. (B) Generation distribution; in red, the terminal branches, in blue the non-terminal, and in pink the intersection between them. (C) Diameter distribution of the terminal branches. (D) Terminal distribution (blue) and density (black dots) along X coordinate. (E) Terminal distribution (blue) and density (black dots) along Y coordinate. (F) Terminal distribution (blue) and density (black dots) along Z coordinate.

Fig 8 gathers an analysis of one of the computed bronchial trees, which is representative of what is observed in other cases. Although we have no experimental information about the exact geometry of minipig bronchial trees, we can analyse the main characteristics of virtual lungs generated and discuss their reliability with existing knowledge and data in general terms. We find a Gaussian distribution around 45° for the bifurcation angular distribution, which is similar to that observed experimentally in humans (Fig 8A). The mean bifurcation angle is 47.7°. The imposition of no angles higher than 90° gives rise to a peak at 90°. As expected, the terminal branch generation is greater than the non-terminal. Most of the branches end at generation 14. The highest generation observed in our computed virtual bronchial trees is between 29 and 31, depending on the dimensions of the lung, which are slightly different for each minipig. The mean terminal diameter is 0.39 mm with a minimum value of 0.1 mm and a maximum of 0.5 mm. The minimum terminal diameter observed is determined by net spacing. Fig 8 also shows the location of terminals and their density along the bronchial tree. This particular minipig’s bronchial tree has 6267 terminals and its volume is 462 cm^3^; this means a density of 13.6 terminals cm^-3^. As shown by black points in Fig 8D–8F, terminal density is isotropic, and thus constant along the 3 spatial axes. This suggests that the bronchial tree is reasonable, because it would be able to supply oxygen to all terminals equally distributed around the pulmonary territory. Terminal distributions show how the pulmonary volume is distributed along the 3 axes (i.e., the lower the pulmonary volume in a certain zone, the fewer the absolute number of terminals).

Most terminals (90%) have the closest terminal between 1.0 mm and 3.2 mm. However, in a few zones with low pulmonary space assigned we may observe great variations in the mean value and each terminal has another terminal at a distance of between 0.1 mm and 6.1 mm.

### Fitting the model with simplest initial assumptions

The updated *Bubble model* is used to simulate the evolution of an initial infection in a computational lung, taking into account experimental results. Experimental CT images showed the final state of the infection; we do not have direct information on its initial location. Coordinates and diameters datasets are analysed to determine whether they follow a Gaussian distribution. These Gaussian distributions would be indicative of a group of initial lesions that were generated from a single infection process and that would have evolved from this common origin with the same mean growth rate. Nevertheless, after a one-sample Kolmogorov-Smirnov test we rejected the null hypothesis, which is that the data were part of a standard normal distribution.

The lack of reliable information about the initial infection entails the need for a blind assumption. Following the law of parsimony, we assume the simplest initial configuration, and alternative possibilities will be explored later on. We use as initial infection for the simulations a single lesion located at the nearest terminal to the mass centre of all the lesions, given the final distribution shown by CT images. The set of parameters is adjusted to minimize the errors as explained in Materials and Methods. followed by the performance of 500 independent simulations of each minipig’s configuration (i.e., a total of 2500 simulations). The set of parameters obtained after the minimization of the objective functions (Eqs 17–19) is *ρ* = 0.13 day^-1^, *β* = 0.08 mm^-1^, and *r*_max_ = 0.68 mm, which corresponds to *NLE* = 0.089, *DE* = 0.283, and *SE* = 0.299.

**Fig 9:**
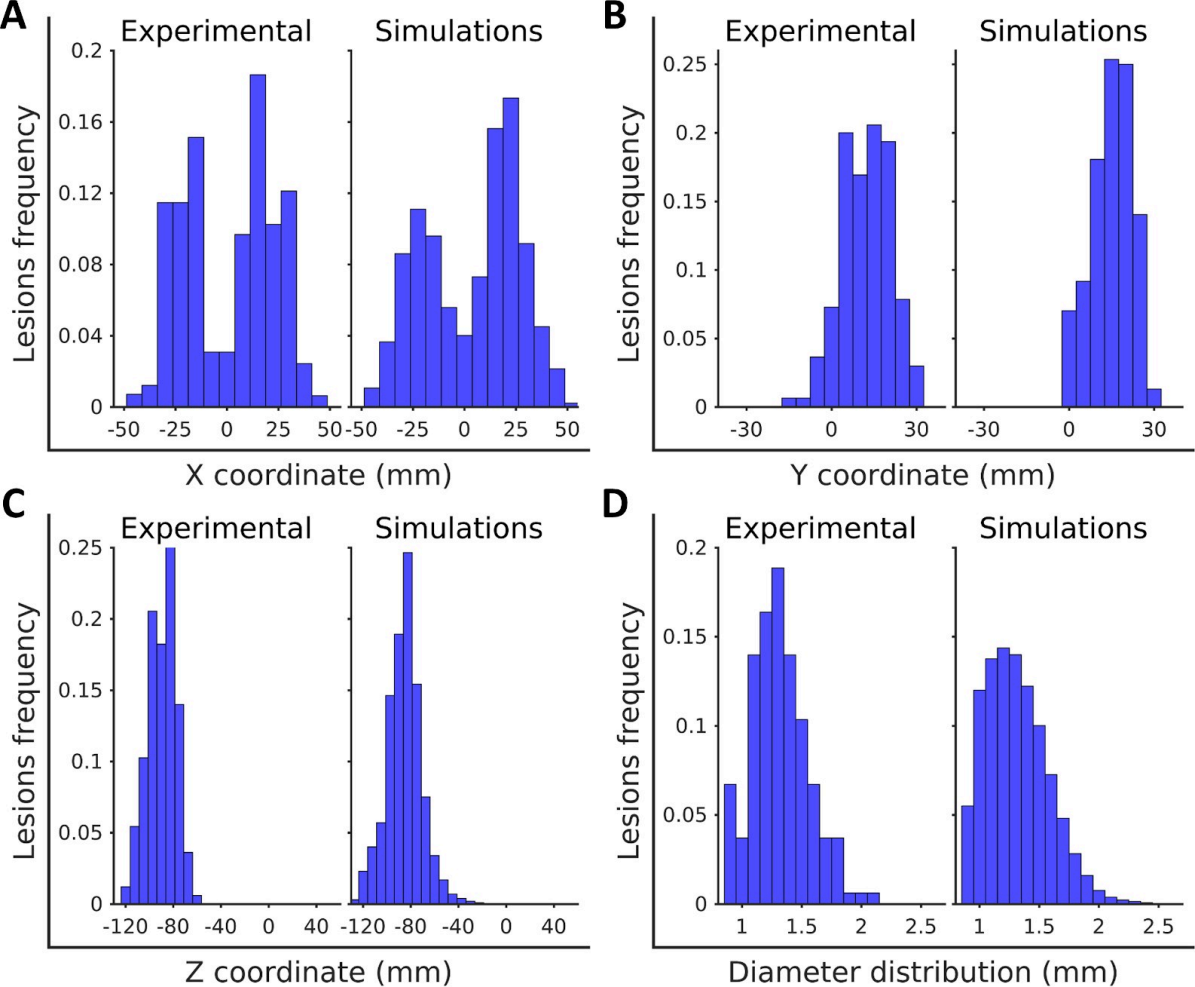
Comparison of experimental and computationally obtained distributions of lesion location and size. Computational distributions were obtained considering as initial infection one lesion in the mass centre of the observed lesions. The set of parameters used is: *ρ* = 0.13 day^-1^, *β* = 0.08 mm^-1^, and *r*_max_ = 0.68 mm. (A) Coordinate X (Left—Right) histogram comparison. (B) Coordinate Y (Anterior–Posterior) histogram comparison. (C) Coordinate Z (Vertical) histogram comparison. (D) Diameter distribution histogram comparison.

In Fig 9 we show the comparison between the experimental and the simulated outcome distributions of spatial coordinates X, Y, and Z, and of lesion diameters. A two-sample Kolmogorov-Smirnov test of the four pairs of distributions show that there are not significative differences in any of the cases with a signification level of 0.05. Therefore, the distributions resulting from the numerical simulation successfully reproduce the experimental observations.

Simulations show that coalescence of lesions is nearly non-existent, on average less than one coalescence per minipig. This result is in agreement with Prats et al. [9] and Marzo et al. [15], who presented coalescence as a mechanism essential to the evolution towards an active disease from a latent infection. A lack of coalescences, therefore, would be a control indicator of the latent infection.

Furthermore, simulation results show that the process of endogenous reinfection is crucial to understanding how lesions appear at different locations in the lungs. The use of a computational bronchial tree for driving such reinfection produces spatial distributions which resemble the experimental cases. This process has been shown to be a key factor in maintaining a latent infection inside big mammals like minipigs.

### Sensitivity analysis

Table 5 shows the results of the sensitivity analysis, with the minimum value for the ANOVA test between the increased and the decreased parameters. This analysis reveals that the number of lesions is strongly related with *ρ*, *ν*, and *T*_max_. It was not obvious that the number of lesions would be related with the growth velocity; however, when lesions grow faster there is an increase in the likelihood that the process of endogenous reinfection will generate new lesions.

**Table 5:** Sensitivity analysis for the set of parameters: S = {*ρ*, *β*, *r*_max_} = {0.12 day^-1^, 0.08 mm^-1^, 0.68 mm}.

		Input parameters				
		*β*	*r*_max_	*ρ*	ν	*T*_max_
Outcome variables	Number of lesions	0.137	0.092	<0.001**	<0.001**	<0.001**
	Mean diameter	0.003**	<0.001**	0.475	0.106	<0.001**
	Dispersion	<0.001**	0.613	0.089	<0.001**	<0.001**
	Coalescences	0.024*	0.004**	<0.001**	<0.001**	0.754

Each value is the minimum of the p-values from ANOVA test comparing 500 runs of the original set of parameters and the set of parameters where one parameter is increased or decreased by 10%. Numbers marked with

* present statistically significant differences with p<0.05 and numbers marked with

** with p<0.01.

The mean diameter varies with parameters *β*, *r*_max_, and *T*_max_. The inflammatory response is the cause of lesion growth, so relations with *r*_max_ and *T*_max_ are expected. The results also show that the dispersion parameter, *β*, slightly affects the mean diameter. A smaller dispersion parameter causes lesions to be closer, thereby increasing the chance of a coalescence event. In fact, as seen in extended sensitivity analysis for the explored parameter space, *r*_max_ and *β* are the two parameters that affect the mean diameter value most. An increase in one of these parameters increases mean diameter value.

According to this analysis, *T*_max_ is the only parameter that is not related with the resulting number of coalescences. All other parameters affect the coalescence processes; nevertheless, a counter-intuitive result is that the dispersion parameter is not the most strongly related. One may expect the dispersion parameter to be the parameter that would affect the coalescence process most because it is the one that determines coalescence spreading, and then determines the distance at which new lesions appear.

The sensitivity analysis evidently depends on the initial set of parameters. Nevertheless, we have employed other sets of parameters to carry out sensitivity analyses, providing equivalent results. These analyses are shown in the Supplementary material section 1 (Table A and Table B).

### Analysing the effect of initial conditions

After confirming that the model with the simplest assumption for the initial conditions is good enough to explain the experimental results, we explored the possibility of improving the agreement between the model and experimental measurements by testing different initial distributions of lesions. Table 6 shows the 12 initial configurations analysed, in addition to the previous one. We explore the choice of one or more lesions from CT data as the initial infection using location, size, and density criteria, as well as different random choices. For each initial distribution, the set of parameters is adjusted to minimize the objective functions as explained in Materials and Methods. Table 6 shows the parameter values that minimize errors as well as the corresponding values for each of the explored initial distributions.

**Table 6:** Initial configurations explored with the model using experimental data.

Initial infection	Parameters set			Errors			
	*ρ* (day^-1^)	*β* (mm^-1^)	*r*_max_ (mm)	*NLE*	DE	*SE*	SE/SE_control_
Mass centre (control)	0.134	0.071	0.67	0.015	0.25	0.30	1.00
Coordinate centre	0.134	0.070	0.66	0.026	0.23	0.34	1.14
Biggest lesion	0.129	0	0.68	0.006	0.18	0.74	2.45
Two biggest lesions	0.102	0	0.68	0.015	0.18	0.73	2.42
30% biggest lesions	0.084	0	0.69	0.009	0.17	0.77	2.58
Densest lesion	0.123	0	0.69	0.003	0.19	0.73	2.43
Two densest lesions	0.110	0	0.68	0.026	0.17	0.73	2.44
30% densest lesions	0.084	0	0.68	0.004	0.16	0.78	2.58
Density>150HU	0.078	0	0.68	0.020	0.17	0.79	2.62
One random lesion	0.227	0.170	0.54	0.027	0.63	0.25	**0.82**
Two random lesions	0.122	0.129	0.58	0.008	0.35	0.23	**0.77**
One random terminal	0.212	0.160	0.55	0.011	0.59	0.71	2.36
Two random terminals	0.129	0.150	0.59	0.016	0.40	0.71	2.37

The first column shows the criteria for choosing which of the measured lesions were assumed as initial infection. The following columns show the parameter values that minimized errors (*ρ*, *β*, *r*_max_) and the values of the three errors obtained (*NLE*, *DE*, and *SE*). The last column compares the spatial error objective function (*SE*) with the one from the control simulation. HU: Houndsfield units.

The results of this analysis, shown in Table 6, do not provide a conclusive criterion for distinguishing those lesions that belonged to the initial infection. Nevertheless, they corroborate that the final lesion distribution is strongly related with the initial infection distribution, since the objective function that is most affected is that of spatial error (*SE*). The distributions that assume as initial infection one or two random experimental lesions provide better spatial error objective function results; however, they give rise to larger values for *DE*.

In some cases, the value of the spatial parameter that minimizes the spatial error function is *β* = 0 mm^-1^. This value permits a macrophage to travel to all other terminals not taking into account the distance through the bronchial tree. In consequence, the final distribution becomes one that follows all terminal spatial distributions and is not related with the particular initial distribution. Therefore, in these cases the initial distribution is related neither with density nor diameter.

### From latent to active tuberculosis: *In silico* experiments

Mathematically we define a case of active disease as one with numerical simulations providing a lesion larger than 1 cm in diameter [35]. The model is designed to reproduce experimental results from latent tuberculosis in minipigs. Therefore, no trigger of disease is observed in any of simulations with the fitted parameters. The following set of simulations is designed to explore the parameter space, looking for those zones leading to active disease.

The parameter space is delimited by *β* ∈ [0, 0.2] mm^-1^, *r*_max_ ∈ [1, 5] mm, and *ρ* ∈ [0.02, 0.2] day^-1^. We used equidistant points, 11 for *β*, 10 for *r*_max_, and 4 for *ρ*. We explored a total of 440 points. We ran 2500 simulations for each point of this parameters space, and 500 for each minipig virtual lung. The initial infection configuration is set as the control (i.e., one lesion in the mass centre of the measured lesions’ distribution). Finally, we define an *Active Disease Index* as the frequency of active cases among the total number of *in silico* experiments for each point of the parameter space.

**Fig 10:**
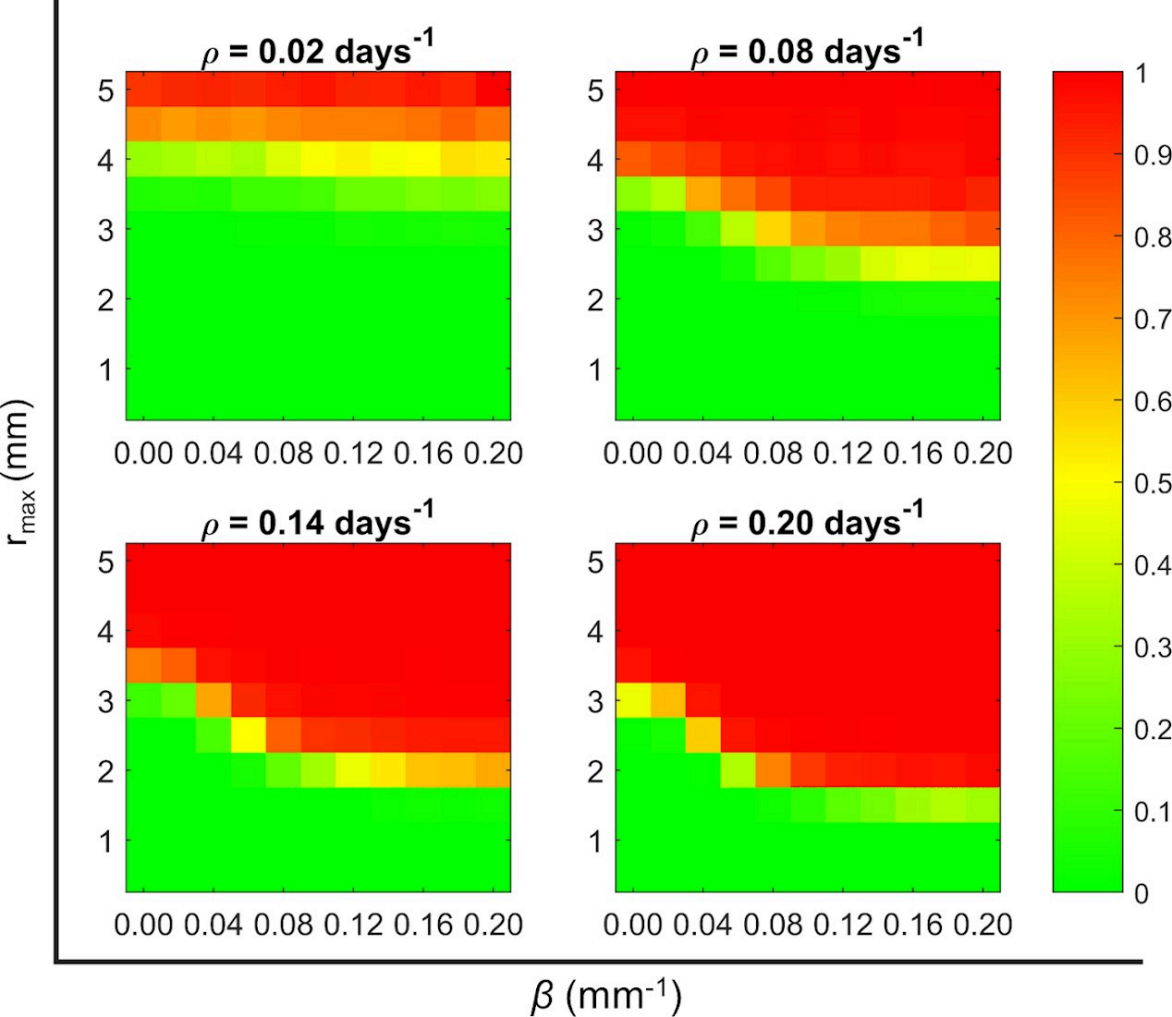
Active Disease Index for different sets of parameters. Exploration of parameters space (*r*_max_, *β* and *ρ*) to see the fraction of *in silico* experiments that present an active TB disease. The colour is proportional to this frequency; green colour means most of the cases remained latent, red colour means that most of the cases derived into an active disease, and intermediate colours mean that both dynamics are possible.

We show in Fig 10 the obtained *Active Disease Index* for each of the explored points. This index increases with any of the 3 parameters, *r*_max_, *β*, and *ρ*. Three different zones of the parameters space can be defined: a latent zone, where most of the simulations result in a latent infection (green colour in Fig 10), an active disease zone, where most of the simulations derive into an active disease (red colour in Fig 10), and a transition zone, where both dynamics are possible (from light green to dark orange in Fig 10).

The parameter *r*_max_ is related with the effective inflammatory response in a broad sense; the greater the effect of the inflammatory response, the bigger the lesions and the greater the likelihood of developing an active disease. This result is in agreement with experimental observations [15] and with other biology systems approaches [14]. Of course, the effective dynamics of inflammatory response can be modulated by local properties such as oxygen concentration or macrophages’ availability, among others, which are not explicitly considered by the model.

Parameter *β* is the dispersion parameter; a higher value of *β* corresponds to lower dispersion inside the lung, which can be a consequence of a lower breathing amplitude. Therefore, a low breathing amplitude appears again as a possible cause for the appearance of big lesions, as was previously described in the literature [9]. It has to be taken into account that breathing amplitude changes from one lobe to another one, i.e., it is wider in lower lobes. Therefore, the conditions that facilitate the development of an ATB would vary from one lobe to another. Our model shows that active disease can be triggered by a high inflammatory response or due to a moderate inflammatory response combined with a small breathing amplitude. Nevertheless, at this point the virtual lungs are considered to be homogeneous, i.e., we are not considering variations of parameters along the lungs’ structure.

After this exploration, the sensitivity analysis is extended to check if sensitivity of the model depends on the set of parameters used. In addition to the default parameters that belong to the latent infection zone, we chose two combinations of parameters: one representative of the active disease zone and the other of the transition zone; see Fig 10. A latent TB parameter set shows that the space parameter, *β*, determines mostly the lesions dispersion; however, a parameter set of the transition zone (*r*_max_ = 6.5 mm, *β* = 0.1 mm^-1^, *ρ* = 0.05 day^-1^) shows that the space parameter affects mostly the number of lesions, mean diameter, and coalescences, and does not affect the dispersion of the lesions. This can be seen in Supplementary material, section 1.

## Discussion

### Limitations and further work

In this modelling approach we have followed the law of parsimony (Occam’s razor) [36], trying to find a simple solution for complex problems such as TB infection dynamics in lungs. The level of complexity was chosen according to the questions to be addressed. This method was developed specifically to submit the main assumptions of the dynamic hypothesis to falsifiability testing. Therefore, the current model includes the most important steps of TB infection evolution suggested by this hypothesis: endogenous reinfection, lesion growth, and coalescence. These processes are supposed to capture the essence of TB dynamics in lungs, but of course they are not the only ones [6]. In fact, no model could be complete [37]. This also allows the use of a fewer number of parameters, when compared with other systems biology approaches to the same problem [1], and thus provides more robustness to the fitting.

The principal novelty of this model is the implementation of the bubble model in an explicit space like the bronchial tree in order to simulate the endogenous reinfection processes. Nevertheless, it still has a few limitations that should be mentioned, the most important being the following:

- Exogenous reinfection is not yet considered in this model. Its incorporation may change the outcome when simulating an ATB infection, as it acts as a new mechanism to generate new infection focuses. Nevertheless, the experimental data used in this study were obtained under conditions that prevented exogenous reinfection. Therefore, the inclusion of this mechanism should be supported by experimental designs that allow it.

- The bronchial tree model is absolutely deterministic, for now. In the future we expect to add some random noise in this algorithm in order to obtain different bronchial trees from a single pulmonary surface. This will be useful for analysing the role of specific bronchial tree properties in TB evolution as well as to account for heterogeneity sources.

- Infection spreading parameters are uniform in each virtual lung. Nevertheless, breathing amplitude is not constant, but varies from lower and middle lobes (wider amplitude) to upper ones (lower amplitude). Breathing amplitude is probably related with lesion spreading; then, higher values of *β* would better fit the local behaviour of less dispersion in the upper lobe, while lower values of *β* would be appropriate for describing the spreading in the lower and middle lobes. This should be taken into account by creating a *β* variable profile inside the bronchial tree. With our model, we have seen that a bigger dispersion reduces the probability of causing big lesions, which is true locally. At the same time, greater dispersion may increase the probability of generating new infection foci in parts of the lung with a smaller breathing amplitude, and therefore with a higher probability of evolving towards bigger granulomas. These dynamics will be carefully explored in the future.

Finally, we must mention the four principal assumptions that could be refined and even refuted in the future:

- Our model follows dynamic hypothesis assumptions [38], but there are more hypotheses that can be submitted to check feasibility such as [39, 40]. In addition, other important processes such as the role of oxygenation [41, 42] could be incorporated.
- The model assumes that the larger the mother, the more likely it is to generate new lesions. This is based on the assumption that bigger lesions would have more foamy macrophages that are more likely to be drained and, therefore, to be able to cause a reinfection [43]. It is also supported by experimental observations in macaques of a higher tendency of bigger lesions to disseminate [44].
- The reinfection model also considers that the older a lesion is, the less likely an infected macrophage is to escapes from it. This is in accordance with experimental observations [22]. In the future, a term that depends on the initial infection time in addition to the age of the lesion could be taken into consideration because, as can be seen in [45], initial lesions are bigger than their daughters in ATB.
- An infection is considered to correspond to an active disease if a lesion grows larger than 1 cm. ATB definition is more complicated than simply having a lesion bigger than 1 cm [35]. Then, other indicators should be explored to define an ATB. In fact, this threshold is not a standard accepted boundary. In supplementary material, section 5 (Fig E in S1 File), an exploration of different values of this threshold is shown. The strength of this model is that the same tendencies that were observed in Fig 10 can be observed in the different exploration threshold simulations.

In general, model falsifiability could be successfully carried out with data from CT (or equivalent) of TB dynamics in big mammals, at different time points. The fact that only a final photo of the system is available is clearly a drawback for the testing of the processes involved. Indeed, tracking the growth of individual lesions at several timepoints should be enough for testing the generalized logistic model. Barcoding techniques have also shown their appropriateness to distinguish contained from disseminated lesions in macaques [44], and thus could provide a way to determine which the initial granulomas are and a pattern of dissemination. Bacteria barcoding, together with a time tracking for the 3D characterization of location and size of lesions by means of CT, can provide key information about the range and relative importance of dissemination, as well as the possible geometrical constraints. Nevertheless, one of the drawbacks of the required experimental tests that should be always kept in mind is that the n is usually small, while the intraspecific diversity is high.

This approach has consisted of the testing of a single model, which seems to go against the strong inference in mathematical modelling [46]. Nevertheless, we have focused on exploring and exploiting all the possibilities given by this model and the available experimental data. In addition to the above-mentioned search for new experimental measurements that can refute the stated hypotheses, future work should include the testing of alternative models whose rejection would provide more clues on the natural history of tuberculosis.

### Conclusions

We built a computational model which includes the virtual lung and the updated *Bubble model*. This model successfully fits CT experimental observations of latent tuberculosis in minipigs. The model incorporates the basis of the dynamic hypothesis [7, 37] to reproduce lesion propagation through the bronchial tree on a latent tuberculosis infection. The agreement between experimental and numerical results reinforces the feasibility of the dynamic hypothesis, i.e., it is able to explain the experimental results observed in latently infected minipigs. In particular, we have observed the importance of the bronchial tree in the endogenous reinfection (*β* > 0).

Most important parameters of the model could be related with the corresponding biophysical processes. Therefore, the model is consistent with the data. Parameter *β* can be related with the breathing amplitude as a factor determining how far new lesions can appear; *r*_max_ may be related with the effect of inflammatory response of the host, as it is the main cause of lesion growth; and *ρ* determines the probability of triggering the endogenous reinfection process. It has been seen that ν, the growth velocity of the lesions, can play a similar role in triggering endogenous reinfection process, while slowing the growth velocity of lesions may be a mechanism for detaining endogenous reinfection.

According to the *in silico* experiments carried out with the model, an active TB in an immunocompetent host may be caused by high inflammatory response or by moderate inflammatory response combined with small breathing amplitude. The former has already been observed experimentally in several studies [15, 21, 22]. The latter may be a clue for understanding the usual presence of active TB in the upper lobe, as suggested by Cardona & Prats [9], since it is in the lung zone that the breathing amplitude is smaller. In fact, further refinements and updates of the model should include inter-lobular differences so that this last possibility can be carefully explored.

## Supporting information

S1 Dataset Experimental dataset of CT measurements. Sheet “Lungs” contains, for each minipig, the pulmonary geometrical measurements and the global distribution of lesions in each lung. Sheet “Lesions” contains the measured characteristics of each lesion.
(XLSX)

S1 File Supplementary material. This file includes the following supplementary sections: (1) Sensitivity analysis for transition and active set; (2) Extended sensitivity analysis; (3) Diameter-length relation in bronchial tree; (4) Details of the simplified protocol for model parametrization sensitivity; and (5) Thresholds exploration.
(PDF)
