# Supplementary material for: Modelling the dynamics of tuberculosis lesions in a virtual lung: Role of the bronchial tree in endogenous reinfection
Source: PLoS Comput Biol. 2020 May 20;16(5):e1007772. doi: 10.1371/journal.pcbi.1007772 (PMC7239440; doi:10.1371/journal.pcbi.1007772)
Supplement: S1 File — This file includes the following supplementary sections: (1) Sensitivity analysis for transition and active set; (2) Extended sensitivity analysis; (3) Diameter-length relation in bronchial tree; (4) Details of the simplified protocol for model parametrization sensitivity; and (5) Thresholds exploration. (PDF) [file pcbi.1007772.s002.pdf]

Modelling the dynamics of tuberculosis lesions in a virtual lung: role of the bronchial tree in endogenous reinfection

Supplementary material

Martí Català, Jordi Bechini, Montserrat Tenesa, Ricardo Pérez, Mariano Moya, Cristina Vilaplana, Joaquim Valls, Sergio Alonso, Daniel López, Pere-Joan Cardona, Clara Prats*

* Corresponding author: clara.prats@upc.edu

Table of contents

1 – Sensitivity analysis for transition and active set	1
2 – Extended sensitivity analysis	3
3 – Diameter-length relation in bronchial tree	7
4 – Details of the simplified protocol for model parametrization sensitivity	8
5 –Thresholds exploration	11
References	13

1. Sensitivity analysis for transition and active set

The sensitivity analysis is performed using as initial infection a single lesion on the mass centre of the experimental lesions observed experimentally in each minipig. The sensitivity analysis is performed for 5 variables: $\beta, r_{max}, \rho, \nu$, and T_{max}. A set of 11 simulations with different parameter values is designed: one simulation with all the parameters at their default values, five simulations increasing one-at-a-time parameter by 10%, and five simulations decreasing one-at-a-time parameter by 10%. We compute an ANOVA test analysis for 4 outcomes by comparing the results obtained with the original set of parameters and those obtained with each new parameter combination [1].

In the main article, sensitivity analysis is performed for latent set of parameters: ρ = 0.12 day^{-1}, β = 0.08 mm^{-1}, and $r_{\max}$ = 0.68 mm (Table 4). In Table A, the results for a transition set of parameters are shown: ρ = 0.045 day^{-1}, β = 0.10 mm^{-1}, and $r_{\max}$ = 6.5 mm. In Table B, the results for an active set of parameters are shown: ρ = 0.045 day^{-1}, β = 0.15 mm^{-1}, and $r_{\max}$ = 10 mm. These tables show that sensitivity analysis depends on the point chosen, but there are some relations that can be observed for the three sets used. There is a dependence between the number of lesions and simulation time ($T_{\max}$). There is also a dependence between mean diameter and β, $r_{\max}$, ν, and $T_{\max}$; dispersion and β show a dependence, as well. Finally, a dependence between the number of coalescences and ρ and ν is found, but no relation with $T_{\max}$.

Other relations are significant in just one or two of the points where the analysis is carried on. For example, in transition and active sets a relation between the number of lesions and β and $r_{\max}$ is found, which is not observed in the latent set.

A point that may be noted is that sensitivity for ρ and ν parameters is almost the same in the three analysed points. Thus, in this model ρ and ν have a very similar role.

Table A. Sensitivity analysis for a transition the set of parameters: S = {ρ, β, r_{max}} = {0.045 day^{-1}, 0.10 mm^{-1}, 6.5 mm}.

		Input parameters				
		β	r_{max}	ρ	ν	T_{max}
Outcome variables	Number of lesions	<0.001**	<0.001**	<0.001**	<0.001**	<0.001**
	Mean diameter	<0.001**	<0.001**	0.134	<0.001**	0.0107*
	Dispersion	<0.001**	<0.001**	0.001**	<0.001**	0.010**
	Coalescences	0.255	0.536	<0.001**	<0.001**	0.251

Each value is the minimum of the p-values from ANOVA test comparing 500 runs of the original set of parameters and the set of parameters where one parameter is increased or decreased by 10%. Numbers marked with * present statistically significant differences with $p<0.05$ and numbers marked with ** with $p<0.01$.

Table B. Sensitivity analysis for an active disease set of parameters: S = {ρ, β, r_{max}} = {0.045 day^{-1}, 0.15 mm^{-1}, 10 mm}.

		Input parameters				
		β	r_{max}	ρ	ν	T_{max}
Outcome variables	Number of lesions	0.005**	0.003**	0.222	0.282	<0.001**
	Mean diameter	0.014**	<0.001**	<0.001**	<0.001**	0.037*
	Dispersion	0.002**	0.013*	0.230	0.746	0.734
	Coalescences	0.004**	0.055	<0.001**	<0.001**	0.257

Each value is the minimum of the p-values from ANOVA test comparing 500 runs of the original set of parameters and the set of parameters where one parameter is increased or decreased by 10%. Numbers marked with * present statistically significant differences with $p<0.05$ and numbers marked with ** with $p<0.01$.

2. Extended sensitivity analysis

An uncertainty and sensitivity analysis is performed for our model as described in [2] using a sampling-based method. Thus, 1000 different parameter sets are used to explore the space using a Latin Hypercube Sampling (LHS) technique. Parameters are explored in the ranges:

$$r_{\max} \in [0, 10] \text{ mm}$$

$$T_{\max} \in [42, 126] \text{ days}$$

$$\beta \in [0, 0.2] \text{ mm}^{-1}$$

$$\rho \in [0, 0.2] \text{ days}^{-1}$$

$$\nu \in [0.15, 0.45] \text{ days}^{-1}$$

$$TH \in [5, 15] \text{ mm}$$

Where TH is the threshold that determines if an infection is considered an Active Tuberculosis (ATB) or a Latent Tuberculosis Infection (LTBI). If there is a lesion of radius greater than TH, simulation outcome is classified as an ATB case. Partial Rank Correlation Coefficient (PRCC) is computed between each of the parameters and outcome parameters (mean and standard deviation): disease indicator, maximum diameter, number of lesions, mean diameter, dispersion, and coalescences. Mean and standard deviation of each outcome are calculated from 1000 simulations of just one minipig. This methodology allowed us to see how each output is affected upon increasing (or decreasing) a specific parameter (linearly discounting the effects of the uncertainty on the rest of the parameters). Thus, PRCC can be used to determine which parameters to target in order to achieve specific goals. Fig A shows a boxplot of the mean value and standard deviation of each outcome variable (first column), the correlation between the mean values and their standard deviation (second column), PRCC values between mean outcome variable value and explored parameters (third column), and PRCC values between standard deviation outcome variable value and explored parameters (fourth column).

Disease indicator increases with $r_{\max}$, ρ, ν, $T_{\max}$, and β, while it decreases with TH. In fact, as can be seen in Fig A, the only significant relation of TH is with disease indicator outcome. This is a coherent result because TH is the threshold that determines whether an infection is considered ATB or not.

Maximum diameter relations are the same as disease indicator relations. This is a coherent result because the disease indicator is directly related with the maximum diameter of the observable lesions.

The parameter that most affects the dispersion is β. Larger values of β entail a decrease in dispersion, as can be seen in Fig 4A of the main article. In fact, in the absence of coalescences the expected PRCC value would be -1. ρ and ν also have a significant relation with dispersion because an increase or decrease in the number of lesions changes the dispersion value, and these two input parameters are strongly related with the number of lesions as can be seen in Fig A(O).

As expected, T_{max}, ρ, and ν are parameters that increase the number of lesions. It can also be seen that β and r_{max} reduce the number of lesions, which is not a trivial result. Larger lesions cause more lesions but also an increase in the probability of coalescence. Thus, the decrease in the number of lesions caused by an increase in β and r_{max} can be explained by the increase in the number of coalescences.

r_{max} is the parameter that has the greatest impact on the mean diameter of the lesions; this is a not surprising result. It can also be observed that an increase in β causes more coalescence, which entails bigger lesions. T_{max}, ρ, and ν have a minor (but positive) effect on the mean diameter due to their correlation with the number of lesions, since more lesions means greater likelihood of finding two lesions closer and then merging creating a new bigger lesion.

Coalescence process is affected by all parameters except TH, as mentioned before. In fact, it is a crucial process that is essential in explaining most of the relations seen in this sensitivity analysis. The two parameters that most affect the coalescence number are ν and ρ due to their relation with the number of lesions.

Disease indicator

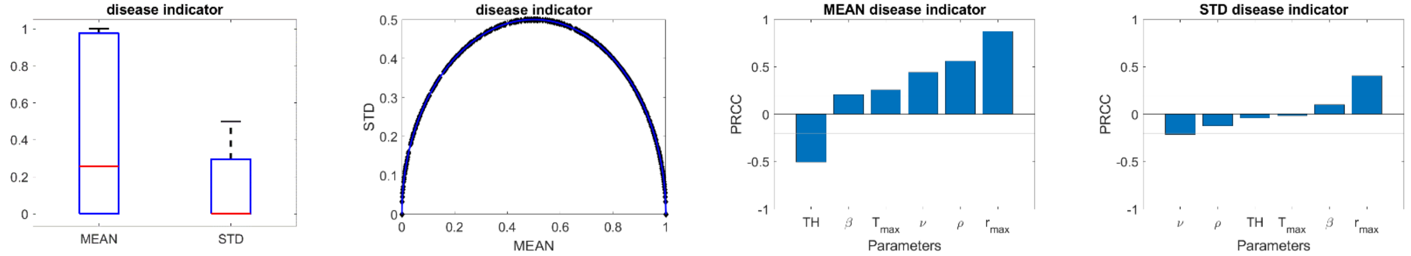

Maximum diameter

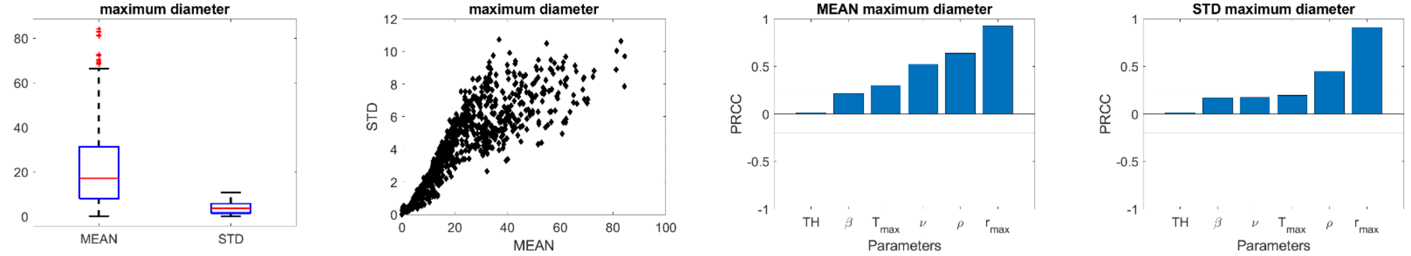

Dispersion

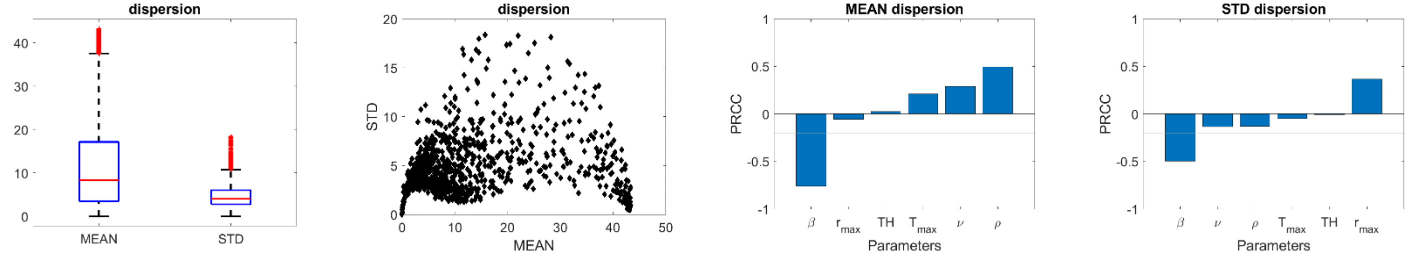

Fig A. Continues on the next page.

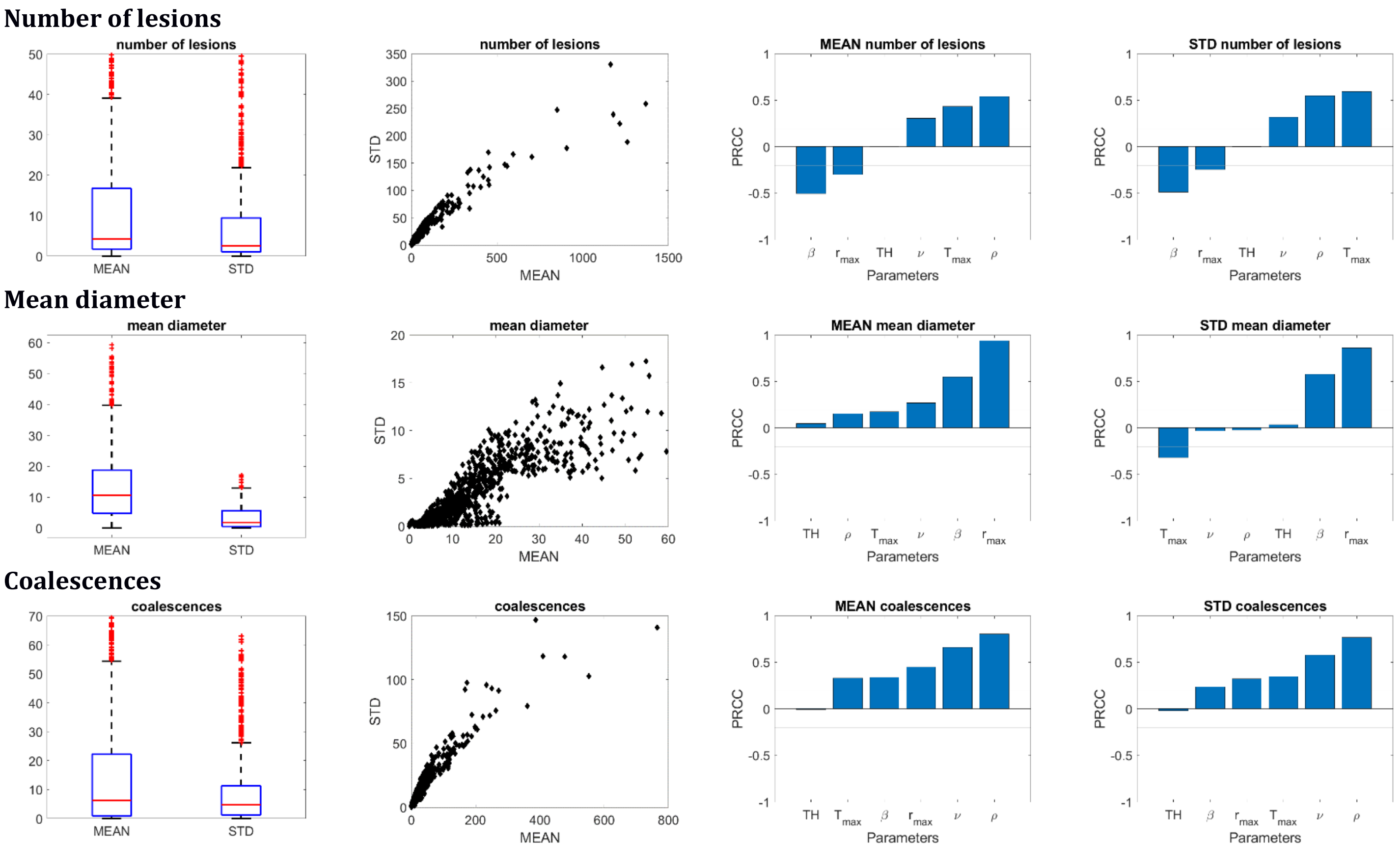

Fig A. Extended sensitivity analysis results. Sensitivity analysis is performed for 6 input parameters (r_{max}, T_{max}, β, ρ, ν, and *TH*) and outcome variables: disease indicator (A-D), maximum diameter (in mm) (E-H), dispersion (in mm) (I-L), number of lesions (M-P), mean diameter (in mm) (Q-T), and coalescences (U-X). First column (A, E, I, M, Q, U): boxplot of mean and standard deviation for each outcome. Second column (B, F, J, N, R, V): relation between standard deviation and mean diameter for each simulation (black point). Third column (C, G, K, O, S, W): PRCC values between the mean values of the outcomes and input parameters. Fourth column (D, H, L, P, T, W): PRCC values between the mean values of the outcomes and input parameters. B: blue line represents the exact relation between standard deviation and mean value as it is a deterministic function.

3. Diameter-length relation in bronchial tree

Although length and diameter of branches in a bronchial tree have a complex relation, a common approximation used in computational bronchial trees is that the length of a certain branch is 3 times its diameter [3, 4].

We used Rozanek and Roubik's [5] data from an adult human bronchial tree to determine the linear relation between diameter and length of the different branches. Using linear regression as shown in Fig B, length and diameter relation can be approximated by:

$$\text{length} = 3.065 \cdot \text{diameter} \qquad \text{(S1)}$$

With a goodness of fit:

$$R^2 = 0.9774 \qquad \text{(S2)}$$

This reinforces the validity of the approximation used in our model.

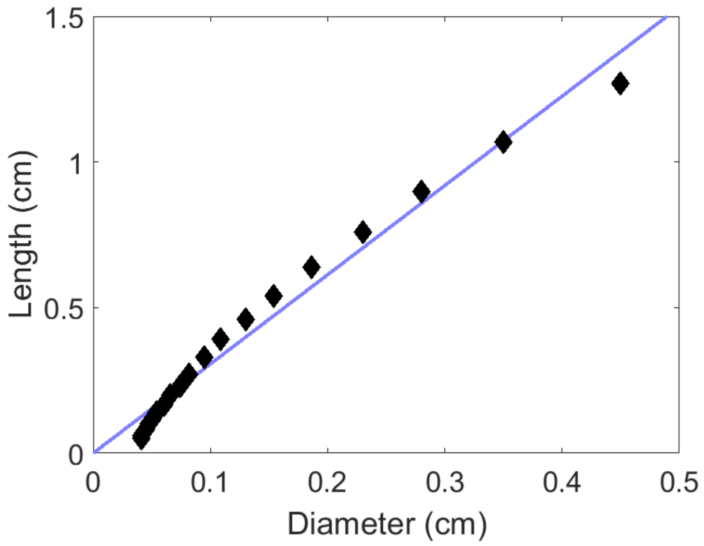

Fig B. Relation between diameter and length. In black, different measurements of branch diameter and length and in blue the linear relation described by the equation (S1).

4. Details of the simplified protocol for model parametrization sensitivity

We aimed to design a protocol for parameterization in order to find the set of parameters (r_{max}, ρ and β) that best fit experimental data. We started our simulations with a single lesion at the mass centre of the experimentally observed distribution. To fit the three remaining parameters, we built three error functions to evaluate the agreement of three outcomes of the computer simulations with experimental data: NLE, DE, and SE, as explained in the main article.

In order to determine how each input parameter affects the defined errors, we ran some simulations. The exploration is done around a desired point: r_{max} = 0.67 mm, β = 0.08 mm^{-1}, and ρ = 0.13 day^{-1}. Two of the parameters are set to their default values and the remaining one is explored. r_{max} is explored between 0.5 and 1 mm, β between 0 and 0.2 mm^{-1}, and ρ between 0.1 and 0.2 day^{-1}.

Fig C shows how errors (NLE, DE, SE) are affected by changes in the input parameters (r_{max}, ρ and β). In Fig C(A,B,C,F,G) the minimum error is clearly seen. These cases correspond to the green cells in Table 3 of the main text. In contrast, Fig C(D) shows that the values change but there is not a clear minimum in the error, which corresponds to the orange cell in Table 3 of the main text. Finally, Fig C(E,H,I) shows no dependence between the error and the input parameter, which corresponds to red cells Table 3.

This analysis is repeated for the midpoint set of parameters as seen in Fig D. Midpoint is: r_{max} = 0.75 mm, β = 0.10 mm^{-1}, and ρ = 0.15 day^{-1}. The obtained results are similar to those shown in Fig C.

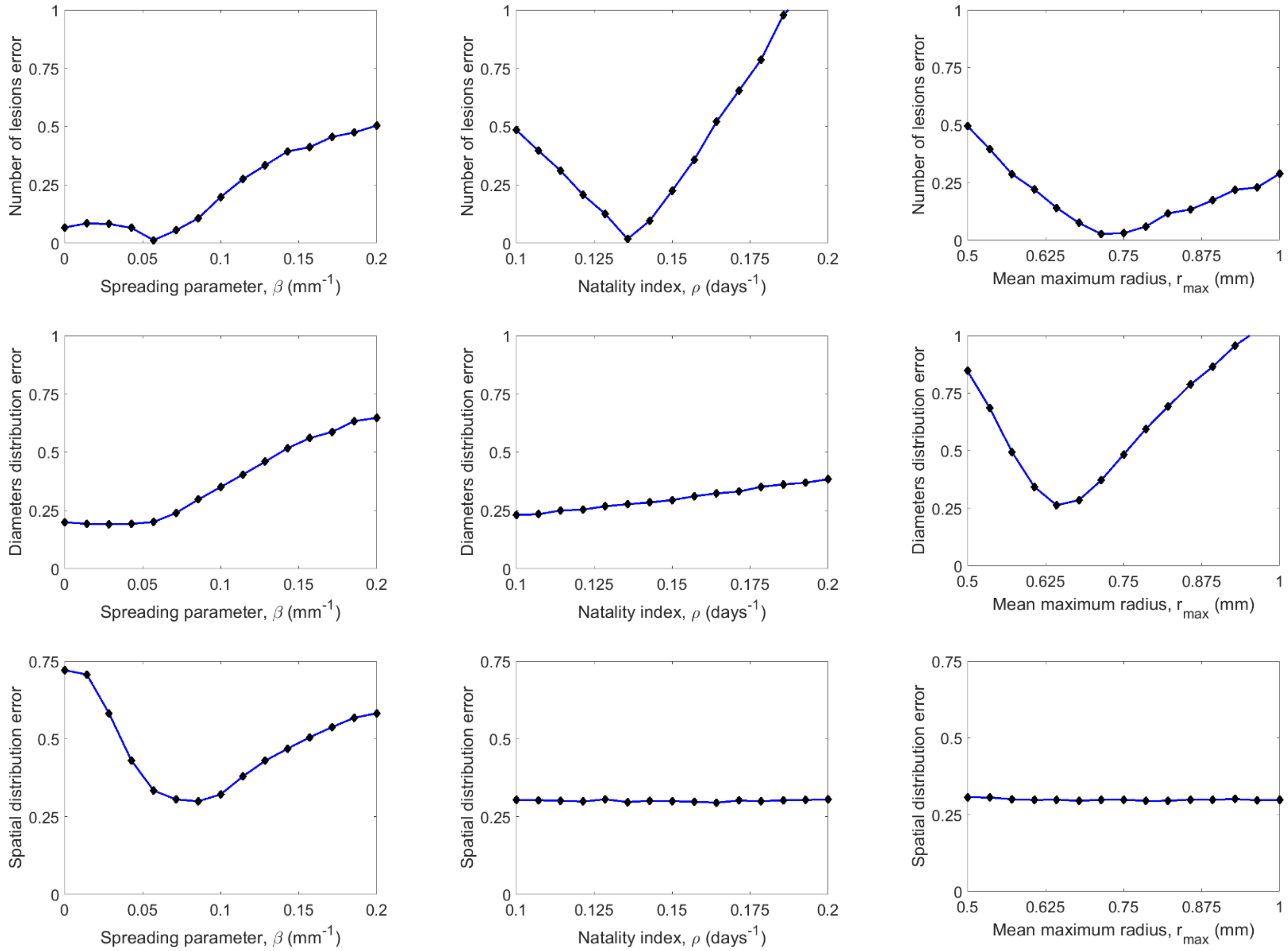

Fig C. Relation between errors and input parameters. The three error functions are number of lesions error (A-C), diameters distribution error (D-F), and spatial distribution error (G-I). These functions are explored for input parameters spreading parameter (A, D, G), natality index (B, E, H), and mean maximum radius (C, F, I) between the ranges: $\beta \in [0, 0.2]$ mm^{-1}, $\rho \in [0.1, 0.2]$ day^{-1}, and $r_{\max} \in [0.5, 1]$ mm. One-at-a-time parameter is explored and the others are fixed at their default values: r_{max} = 0.67 mm, β = 0.08 mm^{-1}, and ρ = 0.13 day^{-1}.

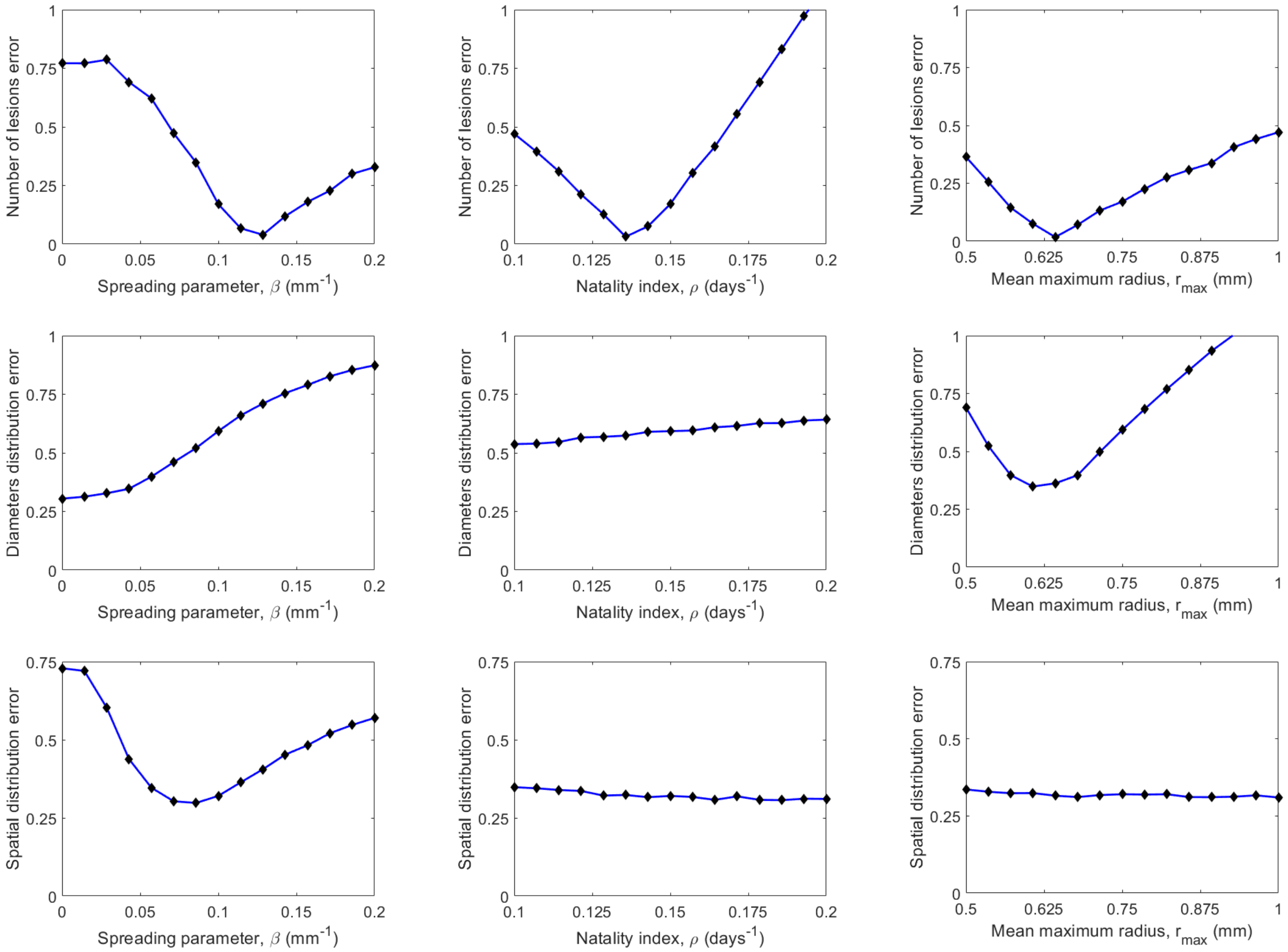

Fig D. Relation between errors and input parameters. The error functions are number of lesions error (A-C), diameters distribution error (D-F), and spatial distribution error (G-I). They are explored for input parameters spreading parameter (A, D, G), natality index (B, E, H), and mean maximum radius (C, F, I) between the ranges: $\beta \in [0, 0.2]\ \mathrm{mm}^{-1}$, $\rho \in [0.1, 0.2]\ \mathrm{day}^{-1}$, and $r_{\max} \in [0.5, 1]$ mm. One-at-a-time parameter is explored and the others are fixed to their default value: $r_{\max}$ = 0.75 mm, β = 0.10 mm^{-1}, and ρ = 0.15 day^{-1}.

5. Threshold exploration

ATB is determined by the biggest lesion. As seen in [6], lesions bigger than 1 cm can be observed in a thorax radiography. Then, threshold radius (TH) is fixed at 5 mm. In Fig 8 of the main article an exploration of the ATB presence (Active Disease Index) with different values of r_{max}, β, and ρ is shown. This analysis is repeated for different thresholds of the disease: lesions bigger than 1, 2, or 3 cm (TH = 5, TH = 10, and TH = 15 mm, respectively). r_{max} is explored between 1 and 10 mm (1 mm separation between simulations), β is explored between 0 and 0.2 mm^{-1} (0.02 mm^{-1} separation between simulations) and ρ is explored between 0.02 and 0.2 day^{-1} (0.06 day^{-1} separation between simulations).

Fig E shows the results of this exploration. The general behaviour is the same as that shown in Fig 8. Our model shows that active disease can be triggered by a high inflammatory response or a moderate inflammatory response combined with a small breathing amplitude. The different thresholds simply move the transition zone towards zones with a higher value of r_{max}.

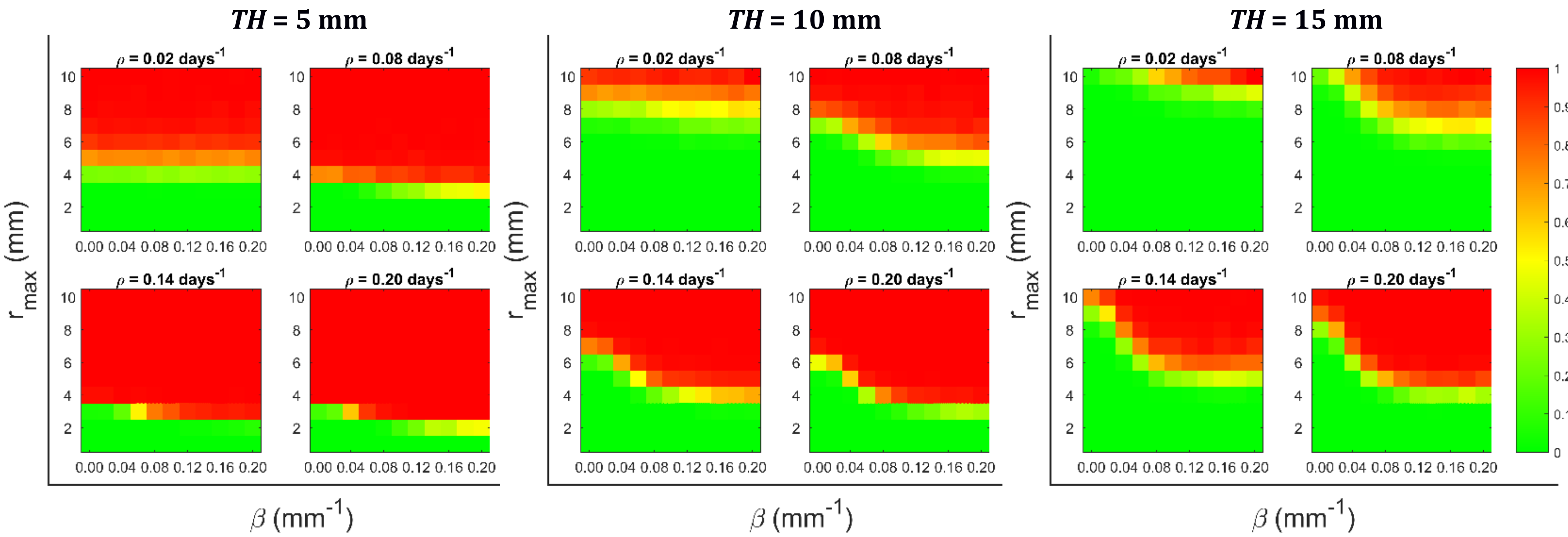

Fig E. Active Disease Index for different sets of parameters (r_{max}, β, ρ, and *TH*). Exploration of parameters' space (r_{max}, β and ρ) to show the fraction of *in silico* experiments that present active tuberculosis disease for different values of ATB threshold (TH). The colour is proportional to this frequency; green means most of the cases remained latent, red means that most of the cases developed into active disease, and intermediate colours mean that both dynamics are possible.

References

1. Ginovart, M., Prats, C., and Portell, X. (2011). “Microbial individual-based models and sensitivity analyses: local and global methods”, in International Conference on Predicitive Modeling in Foods (Dublin), 313–316. Available from: https://upcommons.upc.edu/handle/2117/13546

2. Marino S, Hogue IB, Ray CJ, Kirschner DE. A methodology for performing global uncertainty and sensitivity analysis in systems biology. Journal of theoretical biology. 2008 Sep 7;254(1):178-96.

3. Vegué M. Model tridimensional de l’arbre bronquial humà per a l’estudi de la dissemeniació de Mycobacterium tuberculosis. Master’s Thesis. Universitat de Barcelona. 2012.

4. Kitaoka H, Takaki R, Suki B. A three-dimensional model of the human airway tree. J Appl Physiol (1985). 1999 Dec;87(6):2207-17.

5. Rozanek M, Roubik K. Mathematical model of the respiratory system comparison of the total lung impedance in the adult and neonatal lung. International Journal of Biomedical Sciences 2007; 249-252.

6. Andreu J, Cáceres J, Pallisa E, Martinez-Rodriguez M. Radiological manifestations of pulmonary tuberculosis. Eur J Radiol, 51 (2004), pp. 139-149 doi: 10.1016/j.ejrad.2004.03.009
